# A machine-learning-enabled smart neckband for monitoring dietary intake

**DOI:** 10.1093/pnasnexus/pgae156

**Published:** 2024-05-07

**Authors:** Taewoong Park, Talha Ibn Mahmud, Junsang Lee, Seokkyoon Hong, Jae Young Park, Yuhyun Ji, Taehoo Chang, Jonghun Yi, Min Ku Kim, Rita R Patel, Dong Rip Kim, Young L Kim, Hyowon Lee, Fengqing Zhu, Chi Hwan Lee

**Affiliations:** Weldon School of Biomedical Engineering, Purdue University, West Lafayette, IN 47907, USA; Elmore Family School of Electrical and Computer Engineering, West Lafayette, IN 47907, USA; Weldon School of Biomedical Engineering, Purdue University, West Lafayette, IN 47907, USA; Weldon School of Biomedical Engineering, Purdue University, West Lafayette, IN 47907, USA; Weldon School of Biomedical Engineering, Purdue University, West Lafayette, IN 47907, USA; Weldon School of Biomedical Engineering, Purdue University, West Lafayette, IN 47907, USA; School of Materials Engineering, Purdue University, West Lafayette, IN 47907, USA; School of Mechanical Engineering, Hanyang University, Seoul 04763, Republic of Korea; Weldon School of Biomedical Engineering, Purdue University, West Lafayette, IN 47907, USA; Department of Speech, Language and Hearing Sciences, Indiana University, Bloomington, IN 47408, USA; School of Mechanical Engineering, Hanyang University, Seoul 04763, Republic of Korea; Weldon School of Biomedical Engineering, Purdue University, West Lafayette, IN 47907, USA; Weldon School of Biomedical Engineering, Purdue University, West Lafayette, IN 47907, USA; Center for Implantable Devices, Purdue University, West Lafayette, IN 47907, USA; Elmore Family School of Electrical and Computer Engineering, West Lafayette, IN 47907, USA; Weldon School of Biomedical Engineering, Purdue University, West Lafayette, IN 47907, USA; Elmore Family School of Electrical and Computer Engineering, West Lafayette, IN 47907, USA; School of Materials Engineering, Purdue University, West Lafayette, IN 47907, USA; Center for Implantable Devices, Purdue University, West Lafayette, IN 47907, USA; School of Mechanical Engineering, Purdue University, West Lafayette, IN 47907, USA

**Keywords:** bioelectronics, wearable, machine learning, dietary intake, smart neckband

## Abstract

The increasing need for precise dietary monitoring across various health scenarios has led to innovations in wearable sensing technologies. However, continuously tracking food and fluid intake during daily activities can be complex. In this study, we present a machine-learning-powered smart neckband that features wireless connectivity and a comfortable, foldable design. Initially considered beneficial for managing conditions such as diabetes and obesity by facilitating dietary control, the device's utility extends beyond these applications. It has proved to be valuable for sports enthusiasts, individuals focused on diet control, and general health monitoring. Its wireless connectivity, ergonomic design, and advanced classification capabilities offer a promising solution for overcoming the limitations of traditional dietary tracking methods, highlighting its potential in personalized healthcare and wellness strategies.

**Significance Statement**

This research unveils a cutting-edge smart neckband, leveraging machine learning to facilitate comfortable, noninvasive, and real-time tracking of food and fluid consumption, a vital aspect in controlling chronic conditions, such as diabetes and obesity. In contrast to existing methodologies, our device precisely distinguishes between bodily movements, speech, and eating or drinking events, thereby greatly enhancing the precision of activity categorization, even amid simultaneous activities. Preliminary studies with human subjects validate its effectiveness, positioning it as a noteworthy improvement over current technologies. This innovation is a major leap forward in managing chronic diseases and holds the promise of mitigating related health complications on a global scale.

## Introduction

The escalating demand for meticulous dietary tracking underscores its significance in maintaining comprehensive health and wellness, especially within the spheres of sports nutrition and dietary control (1). This necessity distinctly contributes to advancements in wearable sensing technologies tailored for diverse health contexts. Our proof-of-concept tool offers a versatile solution for accurately tracking food and fluid intake, overcoming the limitations of traditional methods, such as self-journaling and meal photography, which are prone to inaccuracies due to potential over- or underreporting (2, 3). While the tool's utility spans a wide range of applications, it is particularly beneficial for individuals with diabetes or obesity. By providing a reliable means to monitor general food and fluid intake, it aids in the meticulous management of these conditions. For individuals with diabetes, precise tracking assists in optimizing insulin dosage and dietary adjustments, critical for blood sugar control (4). Similarly, for those dealing with obesity, it supports weight management strategies by ensuring dietary intake aligns with nutritional goals. This approach not only facilitates personalized dietary strategies but also enhances disease management by enabling better adherence to recommended dietary practices.

Recent advancements in wearable sensing technologies—equipped with cameras, microphones, piezoelectric sensors, radio frequency modules, and electrophysiological recording units—have made strides in continuously monitoring swallowing events (3, 5, 6). However, these systems face challenges in accuracy, practicality, and reliability, especially when differentiating between bodily movements, speech, and fluid and food intake (5, 7). Classifying food intake within dietary habits is further complicated by factors such as muscle signals, movements, body activities, and environmental conditions present during both fluid and food consumption (3, 8, 9). These complexities significantly impact the accuracy of activity classification, particularly when the wearer is walking. Therefore, there is a continuing need for a solution that is reliable, noninvasive, comfortable, and capable of real-time tracking of food and fluid intake without user intervention. This is crucial for better management of chronic conditions, such as diabetes and obesity (10).

In this study, we introduce a machine learning (ML)-enabled smart neckband, ergonomically designed to comfortably wrap around the neck for continuous monitoring of food and fluid intake throughout daily activities. Engineered for precision, the smart neckband adeptly differentiates among body movements, speech, and fluid and food intake. Its internal sensor module is a custom assembly that harmoniously combines a surface electromyography (sEMG) sensor, a three-axis accelerometer, and a microphone. This configuration is optimized to capture muscle activation patterns in the thyrohyoid muscle of the neck, along with body movements and acoustic signals. Our device leverages a stretchable, twistable, breathable, mesh-structured textile neckband to minimize the common issue of skin delamination seen with small patch devices, offering enhanced user comfort. By avoiding adhesives for an adjustable hook-and-loop fastening system, we ensure a customizable, secure fit, thereby eliminating discomfort and the risk of detachment, making our solution superior for continuous, reliable dietary monitoring and increasing user adherence to monitoring protocols. For data analytics, the smart neckband employs an advanced ML algorithm that combines a random forest (RF) classifier with the Label Powerset algorithm for optimal decision-making (11–14). Pilot studies involving human subjects confirmed its efficacy in continuous monitoring of food and fluid intake, achieving an impressive accuracy rate of 96.04 ± 1.35% for individual activities and 89.26 ± 0.77% for concurrent activities. Recent advancements in soft mechanoacoustic sensors and standalone stretchable device platforms are reflected in the integration of in-sensor adaptive ML, enhancing the accuracy and functionality of wearable technology for activity recognition. As demonstrated in the present work, our device achieves state-of-the-art accuracy for concurrent activities using an RF model (89.26%), with the sEMG, the accelerometer, and the microphone sensors, pointing to future opportunities for further refinement and application in real-world monitoring systems (Table S1).

## Results

### Overall system design with enhanced user comfort

**Fig. 1.**
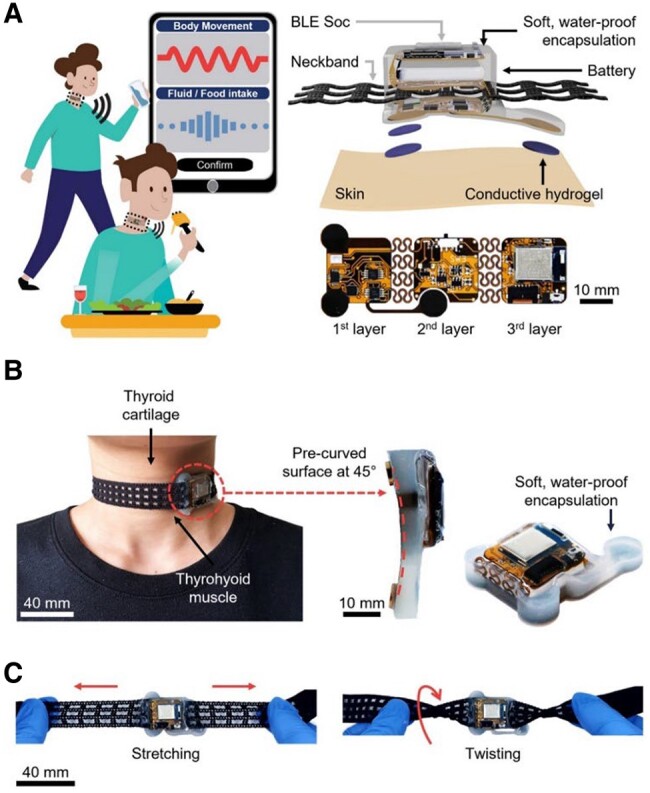
Overall system design with enhanced user comfort. A) Schematic illustration of dietary intake classification via the smart neckband (left). The detailed view showcases a three-layer fPCB, including electronic components on three interconnected layers, serpentine interconnects, the neckband embedded between the first and second layers, and conductive hydrogels for sEMG measurements (right). B) The sensor module, positioned on the thyrohyoid muscle, features a 45° precurved design and soft, waterproof encapsulation. C) Images of the smart neckband being stretched (left) and twisted (right).

Figure 1A presents schematic representations of the smart neckband, ergonomically designed for continuous monitoring of food and fluid intake in both stationary and walking states. Its internal sensor module is constructed using a flexible printed circuit board (fPCB) combined with a total of 47 active and passive components, including: (i) an ultra-low noise microelectronic mechanical system microphone (ICS-40720; TDK InvenSense) to offer a 70-dB signal-to-noise ratio (SNR); (ii) a three-axis digital accelerometer (BMI 160; Bosch) to provide motion measurements with a 1,600 Hz sampling frequency, 16-bit resolution, 0–1,600 Hz bandwidth, and a dynamic range of ±2*g*, where *g* represents the gravitational acceleration of 9.8 m s^−2^; (iii) a custom-designed sEMG amplifier (INA333 and OPA2335; Texas Instruments), featuring a 10- to 400-Hz bandwidth and a gain of 5,000; (iv) a Bluetooth low-energy (BLE) system-on-chip (SoC; nRF52840; Nordic Semiconductor) for data acquisition from the accelerometer, sEMG amplifier, and microphone; (v) a rechargeable 150 mAh lithium-ion polymer battery (ASR00003; TinyCircuits; 20 mm × 20 mm × 5 mm) supported by a charging circuit (MCP73831T; Microchip Technology); (vi) a power management circuit equipped with a regulator (AP2112; Diodes Incorporated) to transit the battery output from 3.7 V to the essential 3.3 V for the sensor module components; and (vii) the conductive hydrogels for sEMG electrodes (RE-D, Electrode Store). Figure S1A displays the complete layout of the fPCB schematic, along with a detailed block diagram of the electronic subsystems depicted in Fig. S1B. This configuration enables wireless data transmission to a smartphone using BLE protocols, as demonstrated in Movie S1. It also offers >18 h of battery life before requiring a recharge, with an average current of 8.08 mA and a battery capacity of 150 mAh.

The internal sensor module of the smart neckband is set in a three-layer, precurved surface at 45°, and shielded by a soft, waterproof elastomer, offering resistance to sweat, and enhanced skin comfort (Fig. 1B). A mesh textile neckband (58673V; Conair) is situated between the first and second layers of the fPCB (Fig. 1A). This is paired with a 10-cm adjustable hook-and-loop strap (VC2 15; Strenco; Fig. S2A). This neckband is designed to fit a range of neck circumferences from 29.9 to 54.0 cm, allowing for a minimal stretch of <15% (15). With the ability to stretch and twist freely, the neckband offers improved comfort and ease of use (Fig. 1C). Commercial neckbands come in various mesh sizes, shapes, colors, and materials, including acrylic, rayon, and polyester, catering to diverse neck sizes and textile preferences (Fig. S2B). The design, which combines the soft-packaged, precurved sensor module with the stretchable mesh textile neckband, aims to minimize the physical mismatch between the smart neckband and the skin, ensuring a comfortable fit (16, 17).

### Mechanical characterizations

**Fig. 2.**
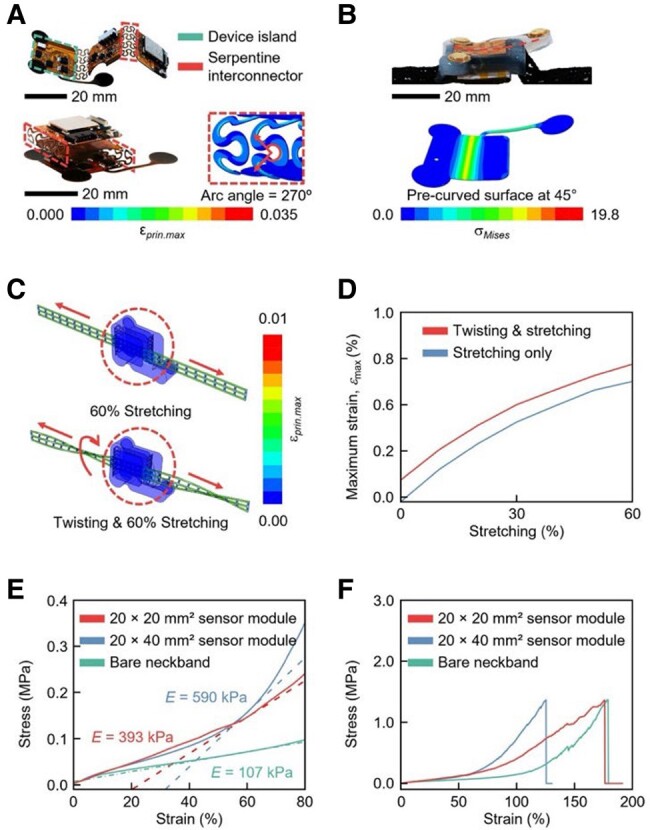
Mechanical characterizations. A) Image of the sensor module (top). FEA results show strain in interconnectors during folding (bottom). B) Image of the sensor module with a 45° precurved design (top). FEA results detail strain on the first layer of the fPCB in this configuration. C) FEA results highlight its mechanical durability under 60% stretching (top) and twisting (bottom). D) FEA data on maximum strain variations in the sensor module under 60% stretching and simultaneous twisting. E) Stress–strain curves for different sensor module sizes and the corresponding bare neckband, displaying mechanical moduli. F) Stress–strain curves for these sensor module sizes and the bare neckband, displaying fracture strain.

Shrinking the internal sensor module in overall size is essential for both precise data collection and user comfort, especially considering the anatomical limitations of the thyroid cartilage and the requirement for accurate placement over the thyrohyoid muscle (3, 8, 17). To achieve this, our design features three 20 mm × 20 mm “device islands” linked by four “serpentine interconnectors,” each with a width of about 200 µm and an arc angle of 270° (18, 19). This arrangement allows the device islands to fold (Fig. 2A, top panel). Electronic components are positioned on these islands for mechanical isolation upon folding (19). Finite-element analysis (FEA) results, shown in Fig. 2A (bottom panel), indicate that the folded, three-layer sensor module undergoes a principal strain not exceeding 3.5% in the folded areas. Movie S2 further illustrates the changes in principal strain as the device islands fold into this three-layer configuration.

Figure 2B highlights the 45° precurved surface of the sensor module, which is designed to reconcile the curvature discrepancy between the module and the natural contour of the neck. Given that neck circumferences can vary widely—from 29.9 to 54.0 cm (15)—a potential curvature gap of 26° to 47° may occur when aligning the sensor module with the skin. This gap could lead to bending stresses, resulting in user discomfort, the potential delamination of the device, or even skin irritation due to excessive pressure (17). Figure S3 presents FEA results comparing sensor modules with 30°, 35°, 40°, and 45° precurved surfaces. When applied to skin curvatures of 26° and 47°, the 30° precurved design exhibited principal strains of 6.5 and 30.0%. In contrast, the 35° precurved design exhibited principal strains of 12.7 and 13.8%, the 40° precurved design showed strains of 13.4 and 7.6%, and the 45° precurved design exhibited strains of only 16.7 and 5.7%, respectively. Additional FEA data, provided in Movie S3, indicate that the sensor module, featuring a 45° precurved configuration, exhibited a von Mises stress of 14.2 MPa and a negligible principal strain of 0.2%. Therefore, the 45° precurved design more closely follows the natural curvature of the neck, effectively reducing the curvature gap and improving mechanical compatibility with the skin.

Figure 2C displays FEA results that illustrate the mechanical durability of the smart neckband under conditions of 60% stretching and simultaneous twisting. These conditions take into account both the up to 29% strain experienced by the skin during dynamic activities and its potential for up to 15% stretch to accommodate variations in neck circumference (20). Movie S4 shows the real-time changes in strain throughout the FEA. Figure 2D presents the corresponding FEA results, which detail the maximum strain variations within the sensor module under these conditions. These findings indicate that the sensor module experiences <1% of the principal strain, highlighting its durability and appropriateness for practical use.

Figure 2E provides a visual representation of the mechanical assessments for the sensor modules in two distinct sizes, 20 × 20 mm^2^ and 20 × 40 mm^2^, when subjected to a 60% stretch. The results demonstrate that the compact sensor module has an elastic modulus of 393 kPa, roughly 50% less than the 590 kPa modulus of the larger one. Moreover, under identical stretching conditions, the smaller sensor module endured a stress of 147 kPa. In contrast, the larger sensor module and the bare neckband recorded stresses of 158 and 72 kPa, respectively. Figure 2F illustrates that, when subjected to strains of up to 120%, the larger sensor module experienced a more significant increase in stress compared with the smaller one. These observations highlight the mechanical benefits and stress resilience of miniaturized sensor modules, particularly in terms of stress alleviation within the smart neckband.

### Comprehensive benchtop evaluations

**Fig. 3.**
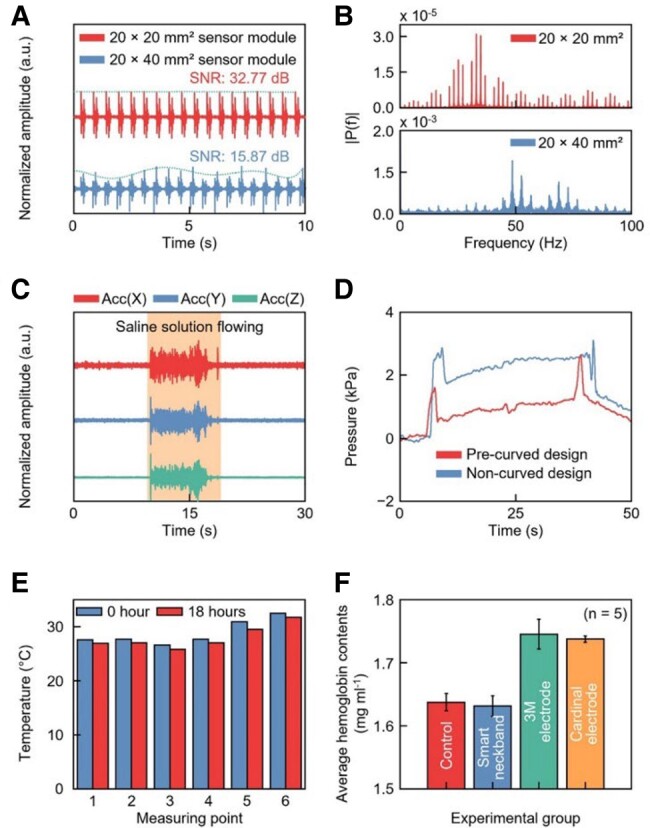
Comprehensive benchtop evaluations. A) Experimental comparison of perpendicular vibration responses across different sensor module sizes. B) Fast Fourier-transformed signals from the sensor modules. C) Tests on the saline waterproofing solution. D) Measurements of skin contact pressure for both precurved and noncurved sensor modules. E) Skin temperature variations over an 18-h period, captured at six measurement points, both surrounding and within the sensor module. F) Average skin hemoglobin content under various conditions: baseline control (bare skin), wearing the smart neckband, and using commercial physiological electrodes (2,560 by 3M and H124SG by Cardinal Health).

To assess the performance of the smart neckband in practical uses, we conducted comprehensive benchtop tests evaluating its response to factors, such as vibration, saline solution exposure, skin contact pressure, temperature fluctuations, and skin irritation. We focused on the typical sub-2 Hz natural vibration frequency of the human body while walking to analyze the signal characteristics from the sensor module under a 2-Hz vibration (8, 21). Figure 3A illustrates that the smaller sensor module (20 × 20 mm^2^) maintained a consistent vibration recording, in stark contrast to the larger module (20 × 40 mm^2^), which displayed irregular waveforms. These dynamics of the real-time vibration tests are further illustrated in Movie S5. We noted the SNR of 32.77 dB for the smaller module, a figure substantially higher than the 15.87 dB recorded for the larger module. Figure 3B presents further insights from a fast Fourier transform (FFT) analysis, revealing a clear 2 Hz signal from the smaller module compared with the substantial noise exhibited by the larger module. The variations in the mechanical resonance frequencies were also notable: around 30 Hz for the smaller module and 50 Hz for the larger module. These variations are attributable to the changes in physical properties as the overall size of the module increases, including a weight increase of 87% from 12.03 to 22.52 g. This enlargement not only necessitates a greater amount of encapsulating polymer but also expands the module area, affecting its elastic modulus. These results underscore the benefits of reducing the module’s size and weight to improve its sensitivity to bodily vibrations.

To verify its resilience to potential sweat exposure during use, we conducted saline solution tests (22, 23). The results, shown in Fig. 3C and Movie S5, demonstrate its sustained functionality under a continuous flow of saline solution (37-6240, McKesson). These tests underscore its reliability in scenarios that are likely to induce sweat, such as prolonged usage, engagement in physical activity, and exposure to high environmental temperatures. We additionally explored the potential pressure-related risks, notably the danger of tissue ischemia, which could occur if the pressure between the sensor and the skin exceeds 4.0 kPa (24). Testing demonstrated that the smart neckband maintains pressures substantially below this threshold—0.97 and 2.49 kPa for the precurved and noncurved sensor modules, respectively (Fig. 3D)—highlighting the ischemia-safe nature of our design, with the precurved format further minimizing skin pressure. Addressing concerns of potential low-temperature burns from prolonged skin exposure (25), our evaluations revealed that the temperature increment peaked at just 1.4 °C throughout an 18-h period of continuous wear, ensuring temperatures did not exceed 31.7 °C (Fig. 3E). Figure S4A offers a detailed visualization of temperature variations on both the sensor module surface and the adjacent skin areas over the 18-h period, attesting to its safety in averting burn risks. Figure 3F presents the measurement results for the average hemoglobin content in the skin, indicative of skin irritation, under various experimental conditions: a baseline control state with nothing attached, a state while wearing the smart neckband, and a state when using commercial physiological electrodes (2560, 3M, and H124SG; Cardinal Health). The results affirm the comprehensive safety of the smart neckband, with no signs of skin irritation noted throughout the testing period. Details of the hemoglobin distribution maps, along with their corresponding RGB (red, green, blue) images, are shown in Fig. S4B.

### Sensor analysis in a stationary state

**Fig. 4.**
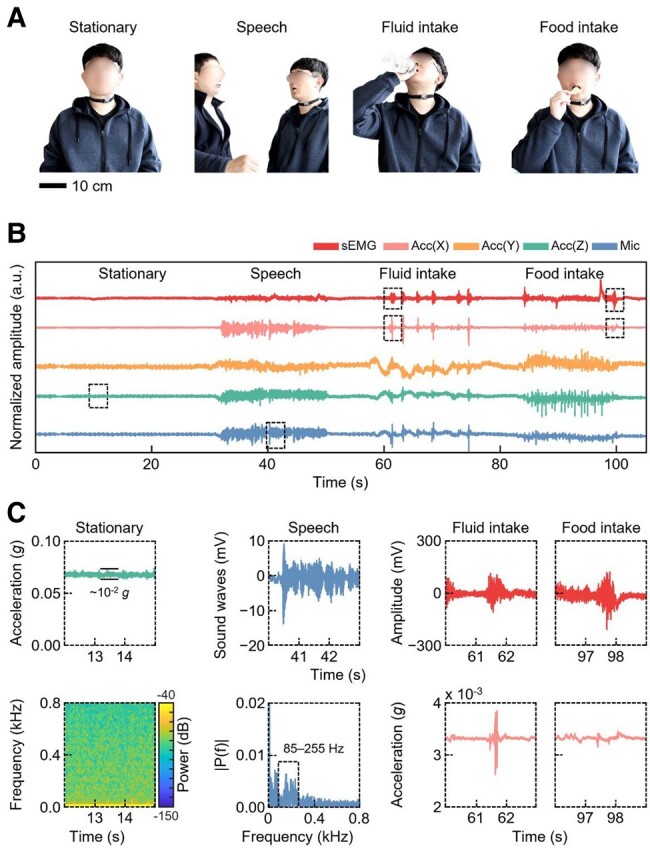
Sensor analysis in a stationary state. A) Photographs of a subject wearing the smart neckband in a stationary state: stationary pose, speaking, fluid intake, and food intake. B) Time series data of sEMG, three-axis acceleration, and microphone recordings captured over a 105-s interval, with the subject engaging in activities such as sitting at rest, speaking, and consuming fluid and food. C) Comprehensive analysis of each activity using a spectrogram for the stationary pose, an FFT for speech, and sensor fusion approaches for fluid and food intake.

Figure 4A depicts a stationary experiment with a healthy 32-y-old male subject who consumes 17 oz of water and potato chips while engaging in activities, such as speaking, and fluid and food intake. Figure 4B illustrates the signals captured by three types of sensors—including the sEMG amplifier, a three-axis accelerometer, and a microphone—strategically positioned on the thyrohyoid muscle. These signals represent the preliminary 5-s baseline state, indicative of a resting phase with no activity, followed by a 20-s stationary state (with natural breathing) before the initiation of each activity. Further data, including details on speech, fluid, and food intake, were collected at 5-s intervals. These measurements clearly delineate distinctive characteristics identifiable in time-domain and frequency-domain analyses, providing a rich dataset concerning deglutition patterns during stationary phases. Figure 4C presents a granular view of the specific signal patterns associated with distinct physiological events. In a stationary state, subtle amplitude signals ∼10^−2^*g* were documented on the *z* axis of acceleration. Addressing speech dynamics, pronounced amplitude signals were observable in sound recordings, with the FFT of these signals showcasing a heightened intensity in the 85–255 Hz frequency band, a characteristic markedly absent in other recorded activities (8). During the investigation into the swallowing activity associated with fluid intake, a burst in signal patterns was observed in both the sEMG and the *x* axis of acceleration. A two-pronged insight was offered: the contraction of the thyrohyoid muscle was captured by the sEMG, while muscle movements occurring during the swallowing process were logged by the *x* axis. This provided a valuable metric for differentiating swallowing activities. Furthermore, the mastication process preceding food intake registered lower amplitude movements in the sEMG, paving the way to a higher frequency ringdown associated with swallowing. This stage marked a distinct shift in the activity spectrum. Furthermore, we investigated the properties of signals produced during the consumption of liquids with varying viscosities, including water and yogurt. Figure S5 illustrates that the consumption of liquids with higher viscosities resulted in a larger magnitude of *y*-axis acceleration, attributable to the augmented exertion necessary for initiating and maintaining the swallowing process. Concurrently, there was a notable increase in the intensity of the low-frequency band within the FFT.

### Sensor analysis in various activities

**Fig. 5.**
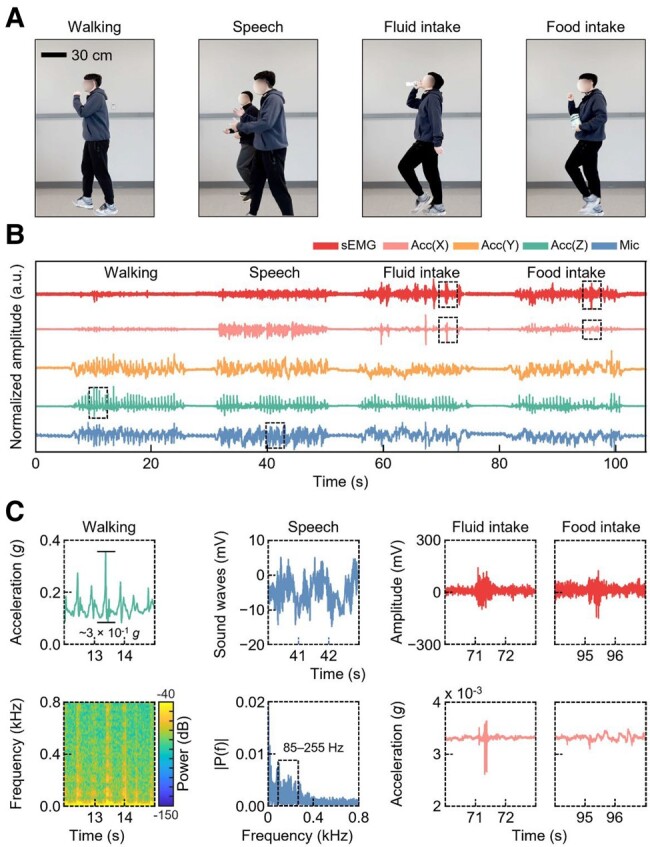
Sensor analysis in various activities. A) Photographs of a subject wearing the smart neckband during various activities: walking, speaking, and fluid and food intake. B) Time series data from sEMG, three-axis acceleration, and microphone recordings over a 105-s span, capturing activities such as walking, speaking, and fluid and food intake. C) Comprehensive analysis of activities using various approaches: a spectrogram for walking, an FFT for speech, and sensor fusion for fluid and food intake.

Figure 5A depicts the experimental setup where the subject was engaged in activities, such as speaking, consuming liquids, and consuming solids while walking around with a steady stride. The activities were carried out under conditions meticulously controlled to mirror those when the subject was stationary, accounting for factors, such as the age of the subject, the type of liquid consumed, and the food ingested. Figure 5B showcases the data captured from the sEMG amplifier, the three-axis accelerometer, and the microphone during this period. This phase of the experiment followed the same sequence as its stationary counterpart. Figure 5C reveals the specific quantitative attributes of the signal patterns associated with individual physiological events during these activities. Upon examining the *z*-axis acceleration when the subject was walking (with natural breathing), we observed signals with larger amplitudes, ∼3 × 10^−1^*g*. The spectrogram analysis corroborates the periodic presence of high-intensity, high-frequency signals under walking states, enhancing our understanding of body dynamics and dietary habits during movement. Regarding speech activities, the sound signals exhibited considerable amplitude fluctuations. Moreover, FFT analyses revealed a higher concentration of intensity within the 85–255 Hz bandwidth compared with other engagements. A notable observation was the marked increase in power within the lower frequency band during walking states compared with stationary phases, a phenomenon likely influenced by movement. Focusing on swallowing during fluid intake, we observed a burst pattern in both the sEMG and *x*-axis acceleration readings, segmented into two unique parts. The first highlighted the contraction of the thyrohyoid muscle, a detail captured by the sEMG, while the second, recorded in the *x*-axis acceleration, showcased the muscle movements occurring throughout the swallowing phase. This distinct signal profile does not appear in data concerning general bodily motions and speech, facilitating precise identification of swallowing maneuvers. When analyzing mastication associated with food intake, initial small-amplitude masticatory actions were detectable in the sEMG readings before the higher frequency ringdown observed during swallowing. We further analyzed the signal properties generated during the walking state while consuming liquids of differing viscosities, including water and yogurt. Figure S6 shows that the ingestion of high-viscosity liquids necessitates enhanced movement during the swallowing process, as evidenced by an increased amplitude of *x*-axis acceleration. Simultaneously, a significant rise in the intensity of the low-frequency bands was detected in the FFT analysis. Importantly, despite the minimal signal magnitudes experienced while attached to the neck, all these signals maintained high reliability, exhibiting SNR values of 13.18 dB for sEMG, 17.18 dB for *x*-axis acceleration, 12.21 dB for *y*-axis acceleration, 15.80 dB for *z*-axis acceleration, and 13.27 dB for the microphone (26–28).

### ML algorithm for activity recognition

**Fig. 6.**
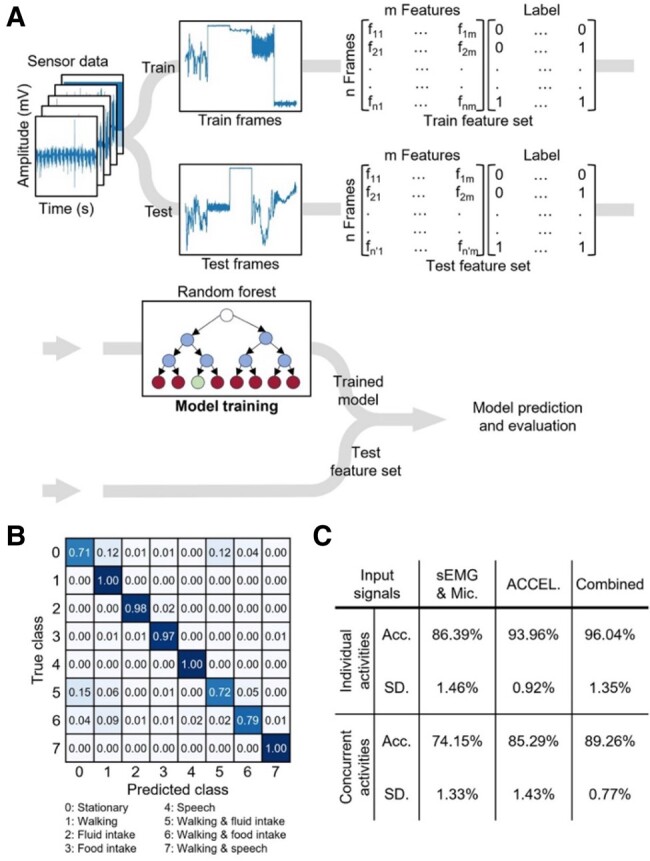
ML algorithm for activity recognition. A) Preprocessed and segmented datasets into frames using a 2-s window. Features were extracted from each frame. Using these features, an RF classifier was trained on the training frames. B) A confusion matrix for classifying eight types of concurrent activities. C) Evaluation of model accuracy based on input signals: sEMG and microphone, three-axis acceleration, and a combination of all inputs. Acc.—Accuracy.

Figure 6A outlines the signal processing pipeline and highlights the critical role the optimization of the parameters of the RF classifier had a significant role in our study. The system utilizes sEMG, three-axis acceleration, and microphone data with the RF classifier and the Label Powerset algorithm for training and prediction, facing challenges in hyperparameter optimization and memory demand. The complexity of managing numerous label combinations requires significant memory, potentially limiting deployment in devices with constrained resources. Optimizing these aspects is crucial for maintaining performance and feasibility. This optimization scrutinized two key parameters: the number of trees in the forest and the frame length, which corresponds to the data capture time window. The number of trees profoundly affects the classifier's efficiency; a forest that is too sparse might overlook essential data patterns, while an overly dense one can risk overfitting, compromising performance on unseen data (29). Through careful calibration using *k*-fold cross-validation (CV) and analyzing between 20 and 100 trees, we determined that a forest of 50 trees offers the optimal balance for peak performance (Fig. S7A). Frame length emerged as another pivotal parameter, with its choice being directly consequential to the amount of information available for classification. A 2-s frame was identified as optimal (Fig. S7B), skillfully avoiding the issues of information scarcity seen with shorter frames and the unnecessary complexity longer frames introduce without substantial performance improvement. Additional fine-tuning was achieved by altering the frame count to assess the classifier’s responsiveness to dataset size, finding the best results with 1,120 frames for individual activities and 940 for concurrent activities (Fig. S7C). Equipped with a refined RF classifier, utilizing 50 trees and a 2-s frame length, we conducted a 10-fold CV on a substantial, healthy training dataset to assess its effectiveness. This classifier, applied to data gathered from subjects performing a wide range of tasks, including both individual and concurrent activities, established a stringent benchmark for our system. Our assessment metrics—subset accuracy and SD, which measure the precise alignment of predicted and true labels and the classifier’s consistency, respectively—revealed an impressive average accuracy rate of 96.04% for individual activities and 89.26% for concurrent activities (Fig. S8). While promising, a more detailed analysis, available in Table S2, is essential to fully appreciate the model's efficacy across various activities.

Figure 6B displays the confusion matrix, offering a detailed account of the accuracy attained for each activity during concurrent activities. A counterpart matrix for individual activities is shown in Fig. S9. In the results of our study, F1 scores for individual activities—stationary (0.859), walking (0.862), fluid intake (0.891), food intake (0.751), and speech (1.00)—demonstrate high classification accuracy, highlighting the effectiveness of our model in distinguishing between these distinct activities (Fig. S10A). For concurrent activities, the model also shows strong performance, with F1 scores for stationary (0.839), walking (0.892), fluid intake (0.891), food intake (0.888), speech (0.9), walking and fluid intake (0.751), walking and food intake (0.796), and walking and speech (0.9), indicating robustness in recognizing complex, simultaneous behaviors (Fig. S10B). In statistical testing, the mean accuracy of our model was found not to be significantly different from the hypothesized value of 0.97 (*P* = 0.0599; *T* = −2.151). Furthermore, Cohen's kappa scores for individual activities (0.9475) and concurrent activities (0.8827) confirm an almost perfect level of agreement in the classification tasks.

These matrices affirm the high precision with which our model can categorize both complex individual and concurrent activities. We extended our analysis to evaluate the impact of various input signals on the efficiency of the model, which leveraged inputs including audio, sEMG, and data from a three-axis accelerometer. Notably, the accelerometer data emerged as a pivotal factor in predicting both individual and concurrent activities, underlining its central role in activity recognition (Fig. 6C). This underscores the significance of integrating audio, sEMG, and accelerometer data to boost the performance of activity recognition systems. Hyperparameter adjustments, especially in the number of trees, have a notable impact on our model's performance, reflecting meaningful improvements across different activity types. For individual activities, the accuracy enhancement of 2% (from 0.9396 to 0.9604) not only demonstrates statistical significance but also translates into practical reliability in real-world applications. For concurrent activities, the 4% improvement in accuracy (from 0.8529 to 0.8926) and the associated decrease in SD (−0.66%) highlight the model’s enhanced capability to handle complex scenarios, underscoring the practical significance of tailored hyperparameter tuning. In the RF model, diversity was introduced by training each decision tree on a random subset of data and features, enabling the model to capture the unique characteristics of concurrent activities. Despite some overlap, activities such as fluid intake and walking possess distinct features, allowing the RF algorithm to accurately differentiate and predict these complex behaviors. Additionally, we conducted a comparative analysis, juxtaposing our approach with those documented in other studies, as also summarized in Table S1. Our methodology not only excels in offering the most pragmatic form factor for real-world application, achieving unmatched accuracy for individual activities but also successfully realizes high precision in distinguishing concurrent activities, a milestone yet unreached in other research initiatives.

## Discussion

### Limitations of the study

In the pursuit of advancing dietary monitoring technologies, our smart neckband represents a significant step forward, although it is accompanied by certain constraints that provide opportunities for future research. While the device is designed with an adjustable strap to accommodate various neck circumferences, ensuring comfort across diverse body types remains an ongoing endeavor. Feedback from a wider user base will inform iterative enhancements to meet a broader range of personal preferences and ergonomic requirements. Additionally, the current scope of testing did not include data from patients with diabetes or obesity. Future studies are anticipated to bridge this gap, enhancing the device's algorithmic accuracy through exposure to more complex dietary patterns and management needs specific to these populations. The types of food that the device has been tested with are currently limited, but this represents a starting point from which the device can learn and adapt, with plans to expand the range in subsequent research phases. While a closed-loop system integrating continuous glucose monitors (CGMs) and insulin pumps for comprehensive dietary management based on blood sugar levels is not yet within the scope of the current model, this concept opens an avenue for multidisciplinary collaboration and technological innovation.

## Conclusion

This study explores the potential of a smart neckband equipped with an sEMG sensor, a three-axis accelerometer, and a microphone sensor for monitoring food and liquid intake. Positioned on the thyrohyoid muscle, the neckband aims to monitor deglutition patterns by analyzing the data collected through ML algorithms, specifically an RF classifier. Featuring a foldable, precurved design and constructed from breathable mesh textile, the neckband is meticulously designed for comfort and efficient signal capture. It underwent rigorous testing for various parameters, including vibration sensitivity, sweat resistance, and its impact on skin pressure, temperature, and irritation, thereby demonstrating its suitability for prolonged use. The device holds promise for aiding in behavioral modifications essential for weight management and is versatile enough to support a wide range of applications, including sports nutrition and general health improvement.

It holds the potential to analyze eating habits and physical movements, which can be synthesized into actionable insights through a prospective smartphone healthcare app, thereby encouraging healthier behaviors. For instance, identifying patterns, such as frequent eating while walking, could inform personalized interventions. This capability extends beyond traditional health management to encompass applications in sports performance and daily wellness, highlighting its utility as a proof-of-concept tool for broader dietary monitoring. Importantly, the device's potential to assist in calculating insulin dosages for diabetic patients, by identifying meal timings, exemplifies its specific benefit in disease management. Its primary function—facilitating a comprehensive understanding of an individual's dietary and activity patterns—makes it an invaluable tool for anyone seeking to optimize their health and dietary habits, particularly those with diabetes or obesity. Future integration with CGMs could offer a more comprehensive perspective on blood sugar management, enhance guidance for insulin dosing, and deepen our understanding of the relationship between food intake and glycemic levels (30). Currently, challenges persist, especially concerning the uncertainties of meal timing in the context of insulin administration for diabetic patients (31). While CGMs are adept at tracking insulin usage, they do not elucidate the reasoning behind specific doses. The neckband's capability to identify meal occasions might help fill this gap, potentially leading to more precise insulin dosing guidelines. Finally, this study establishes a solid foundation for further research and development in dietary monitoring and general health management. It signals a move toward more informed and personalized healthcare solutions, with the neckband serving as a novel dietary monitoring wearable tool to enhance data interpretation and inform behavioral modifications, supporting a broad spectrum of health and wellness goals.

## Materials and methods

### Fabrication of the smart neckband

Commercial software (Autodesk Eagle Version 9.6.2) was used to generate schematic diagrams and layouts for the fPCB. The smart neckband was designed with a three-layer-stacked, three-island fPCB, incorporating commercially available electronic components. The first island housed the three-axis digital accelerometer, a custom-designed sEMG amplifier, and a microphone. The circuits responsible for battery charging and voltage regulation were located on the second island, while the third island housed the BLE SoC and LEDs. The thyrohyoid sEMG signal measurements employed a small diameter (6 mm) and interelectrode distance (20 mm) to reduce detection volume and minimize crosstalk effects, consistent with the sEMG for the noninvasive assessment of muscles (Seniam) guidelines (32). Customized firmware was uploaded to the BLE SoC. After the various surface-mount components were placed, they were soldered to the fPCB, and the system was folded, marking the completion of the electronics’ fabrication. Solder paste (SMDLTLFP10T5; Chip Quik) was used to attach the various surface-mount components to the fPCB, facilitated by a heat gun (Int866; Aqyue) and hot plate (MHP30; Miniware). Once soldering was completed, the fPCB was covered with a silicon conformal coating (422C-55MLCA; MG Chemicals) to strengthen the solder bonding (Fig. S11). An off-the-shelf mesh textile neckband (58673V; Conair) was then placed between the first and second layers of the fPCB to integrate the neckband. The entire structure was subsequently encapsulated with a soft, waterproof elastomer (Ecoflex 00-35; Smooth-On) to shield the system from external factors and offer a sturdy, long-lasting interface for manipulation. To guarantee a secure attachment of the neckband to the skin, the skin interface layer was made of a conductive hydrogel (RE-D; Electrode Store) for the sEMG electrodes (Fig. S12). This layer was shaped using a 6-mm round hole punch (K003; Kucaa) to match the electrode layout of the neckband.

### Encapsulation of the sensor module

A mold was designed using commercial 3D CAD software (Autodesk Fusion 360 Version 2.0) and was printed using a stereolithography 3D printer (Form3; Formlabs). Before starting the encapsulation process, a silicone release agent (Ease Release 200; Mann Release Technologies) was applied. The encapsulation was carried out with a fully fabricated sensor module and a textile neckband positioned within the mold. A soft elastomer gel (Ecoflex 00-35; Smooth-On) was utilized as the encapsulation layer.

### Characterization of mechanical reliability

Mechanical analysis was conducted using FEA through a commercial software package, Abaqus, to investigate the stress and strain levels applied to the sensor module by folding, bending, stretching, and twisting, as well as the interfacial strain on human skin when the sensor module was attached. The developed sensor module was modeled, comprising a polyimide (PI) frame covered with an Ecoflex 00-35 body through which a textile line passed. Linear elasticity was applied to the PI frame, brass alloy electrode, and human skin with elastic moduli of 7.1 GPa, 97 GPa, and 10 kPa, and Poisson's ratios of 0.30, 0.31, and 0.48, respectively. A hyperfoam material model was applied to the textile based on uniaxial testing data. A Neo-Hookean hyperelastic model was applied to Ecoflex 00-35 with coefficients, C10 of 0.0113 and D1 of 1.96. Displacement and rotation boundary conditions were set for the edges of the PI frame, surfaces of Ecoflex 00-35, and the electrode. An embedded region constraint was applied to the PI frame, textile, and brass alloy electrode with Ecoflex 00-35 selected as the host material. Self-contact interaction was set for the textile, and surface contact interaction was set for the bottom surfaces of the brass alloy electrodes with the skin surface to analyze interfacial deformation. Subsequent to the computational work, an experimental study was conducted using a motorized force tester (ESM303, Mark-10), where the smart neckband was subjected to stretching conditions.

### Comprehensive benchtop tests

In analyzing the inherent vibrational response of the sensor module, we used a vibration generator (1000701; 3B Scientific) and an arbitrary waveform generator (3390; Keithley) to produce the targeted vibration. The waveform parameters were set to 2 Hz, square wave, 4 *V*_Peak-Peak_, with an 80% duty cycle. To assess the sweatproof performance of the sensor module, we repeatedly exposed it to external 0.9% saline solution (37-6240; McKesson) for 20 s. The sensor module was observed functioning normally under flowing saline solution, successfully transmitting sensor data via Bluetooth. To measure the pressure between the skin and the sensor module, we evaluated both the precurved and noncurved sensor modules using a miniaturized pressure sensor (CSU8-1N; SingleTact). The impedance assessment, shown in Fig. S13A, was conducted using a potentiostat (SP-200; BioLogic) in a two-electrode setup, spaced 2.5 cm apart center-to-center. For comparative purposes, we used commercial Ag/AgCl electrodes as both the reference/counter electrode (RE/CE) and the working electrode. These were evaluated against the sEMG electrode integrated into the sensor module. The surface area of the commercial Ag/AgCl electrode was 1.77 cm^2^, while that of the sensor module electrode was 0.28 cm^2^. Additionally, through experimental validation, we confirmed the variations in impedance observed when the hydrogel electrode was exposed to air for intervals of 0, 3, 6, and 9 h (Fig. S13B). All tests were performed on the thyrohyoid muscle, using potentiometric mode and applying a sinusoidal signal with a 1-mV amplitude. The frequency range for the measurements spanned from 1 to 800 Hz (33). To monitor temperature changes when the sensor module was operational on the skin for 18 h, we used a high-resolution science grade long-wave infrared camera (A655sc; FLIR).

### RF classifier methodology

The RF classifier was employed to automatically identify a range of activities. This tool constructs multiple decision trees from different subsets of the dataset and amalgamates their predictions to determine a final outcome. Its versatility allows it to detect complex patterns in the data, rendering it ideal for multifaceted classification tasks. The RF classifier was fine-tuned using *k*-fold CV, wherein 90% of the data were allocated for training and the remaining 10% for testing in each iteration, a process repeated for 10 iterations. The classification results were then averaged across these iterations. For the individual activities, the total dataset size was 5,600 frames. For concurrent activities, the total dataset size was set to 7,520 frames. Following the sampling and subsequent concatenation of the five signals, the final dimension for each frame was established at (1, 19,000).

### Human subject study

This study involved healthy volunteers and was conducted with Institutional Review Board (IRB) approvals (#IRB-2023-1002 and #HYUIRB-202212-009-3) at Purdue University and Hanyang University. The participant pool consisted of six volunteers, with an age range of 29–34 years and an equal distribution of male and female subjects. Selection criteria were as follows: (i) being aged between 18 and 55 years, (ii) the capacity to understand and give informed consent, and (iii) a willingness to fully participate in the study procedures, including wearing the wearable device for the activity-rest cycles. Prior to the main procedures, participants attended an orientation session to familiarize themselves with the study's expectations. An eligibility survey was administered to determine if participants met the study criteria. Those who qualified received a comprehensive consent form outlining the study's objectives, participant responsibilities, and ethical considerations, such as potential risks and benefits. Before wearing the smart neckband, participants were briefed on its purpose and functionality. The sensor module was fitted on each participant's thyrohyoid muscle. The experimental protocol consisted of alternating cycles of 20-s rest and 20-s activity intervals, repeated 20 times for an approximate total of 13 min. This cycle was conducted four times, each for different activities: body movement, fluid intake, food intake, and speech. Thus, each session took an estimated 54 min to complete. The procedure described above was carried out under two conditions: stationary and walking (striding at a comfortable pace), with the total duration being ∼108 min per session.

## Supplementary Material

pgae156_Supplementary_Data

## Data Availability

Modified data that support the plots and other findings of this study are available at https://www.kaggle.com/datasets/tibnmahm/1d-signal-collection-for-dietary-intake-analysis.
