# Supplementary material for: A machine-learning-enabled smart neckband for monitoring dietary intake
Source: PNAS Nexus. 2024 May 7;3(5):pgae156. doi: 10.1093/pnasnexus/pgae156 (PMC11075646; doi:10.1093/pnasnexus/pgae156)
Supplement: pgae156_Supplementary_Data [file pgae156_supplementary_data.zip › PNASNEXUS-PNASNEXUS-2023-01256-TRR-s01.docx]

**Supporting Information for**

A machine learning-enabled smart neckband for monitoring dietary intake

Taewoong Park^a,1^, Talha Ibn Mahmud^b,1^, Junsang Lee^a^, Seokkyoon Hong^a^, Jae Young Park^a^, Yuhyun Ji^a^, Taehoo Chang^c^, Jonghun Yi^d^, Min Ku Kim^a^, Rita R. Patel^e^, Dong Rip Kim^d^, Young L. Kim^a^, Hyowon Lee^a,g^, Fengqing Zhu^b,^*, Chi Hwan Lee^a,c,f,g,^*

^1^T.P., T.I.M. contributed equally to this work.

*Corresponding author name(s): Fengqing Zhu and Chi Hwan Lee

Email: zhu0@purdue.edu (F.Z); lee2270@purdue.edu (C.H.L.)

**This PDF file includes:**

Supporting texts 1 to 6

Figures S1 to S14

Tables S1 to S3

Legends for Movies S1 to S5

SI References

**Other supporting materials for this manuscript include the following:**

Movies S1 to S5

Supporting Information Text

**Supporting Text 1. Optimized sensor placement and miniaturization.** The swallowing process, which involved complex orchestration among numerous muscles such as the suprahyoid, pharyngeal constrictor, infrahyoid, and cricopharyngeus muscles, garnered attention due to the valuable insights it could provide into dietary intake data (1). However, the intricate positioning and movement of these muscles during the swallowing process presented significant challenges in the accurate deployment of wearable sensors for precise activity tracking. A proposed solution was the placement of the smart neckband on the thyrohyoid muscle, a member of the infrahyoid muscle group, situated superiorly to the laryngeal prominence and adjacent to the thyroid cartilage. The close proximity and origination of the thyrohyoid muscle from the lamina of the thyroid cartilage made it an ideal site for swallowing activity monitoring. However, implementing this strategy came with a challenge. In the context of thyroid cartilage anatomy, the length of this structure is notably diminutive (2, 3), necessitating a compact wearable device to measure the swallowing signal from the adjacent thyrohyoid muscle. To accurately position the device on the thyrohyoid muscle, miniaturization of the device was essential. In response to this need, we developed an advanced foldable design that reduced the size of the smart neckband to 20 mm × 20 mm × 9 mm with the fPCB.

**Supporting Text 2. Sensor data analysis.** All analyses were conducted using the MATLAB (R2021b) technical computing language. The digital manipulation phase involved the use of a fourth-order Butterworth infinite impulse response filter, which was followed by an anti-causal, zero-phase filtering approach implemented through the MATLAB 'filtfilt' function. To illustrate the distribution of signal frequencies over time, we undertook spectrogram analysis using the MATLAB 'pspectrum' function. Moreover, we executed FFT analysis using the MATLAB ‘FFT’ function to identify the individual periodic components of the signal, aiding in a deeper comprehension of the signal characteristics. Exploring the details of signal processing, the onset of swallowing events was marked by slow movements (~0.1 s) of the vocal folds and larynx mechanics during the pharyngeal phase. This phase ended with a high-frequency ringdown associated with the flow of fluid or food during the esophageal phase. The swallowing events encompassed both low-frequency mechanical motions (0.1–5 Hz) and high-frequency acoustic components (100–800 Hz). We also noted the complexity of mastication biomechanics, where the mandibular cycle typically exhibited signal frequencies in the 1–2 Hz range. Speech signals were distinct, displaying rich harmonic structures with fundamental frequencies that generally ranged between 85 and 255 Hz for the adult population.

**Supporting Text 3. SNR calculation.** The SNR was compared using power spectral density estimation in MATLAB. The SNR in decibels (dB) was calculated using the following formula:

$\text{SNR}_{\text{dB}}\text{=10}\text{log}_{\text{10}} \left( \frac{\text{P}_{\text{signal}}}{\text{P}_{\text{noise}}} \right)$ Eq. S1

where $P_{signal}$ is power of signal and $P_{noise}$ is power of noise.

**Supporting Text 4. Skin irritation tests.** Inflammation accompanied by erythema, which changes the concentrations of hemoglobin, is a common response after irritation of the human skin. To quantify the inflammation caused by irritants, we used a line-scan hyperspectral imaging system. The hyperspectral imaging system is to acquire a hyperspectral image (hypercube) of a sample to analyze spectral fingerprints from a specific area (4, 5). The line-scan hyperspectral imaging system we used has a slit that had a width of 23 µm. The light passing through the slit was dispersed by a diffraction grating (groove density = 150 mm^-1^) and captured using a monochrome camera (GS3-U3-120S6M-C, FLIR). An LED light source with a color temperature of 6500K (D65) was used as the illumination source. Spectral calibration of the spectrograph was performed using a xenon calibration light source that emits multiple narrow peaks at specific wavelengths. A fixed focal length lens (MVL25M1, Navitar) was used mainly to image the skin area and the field of view was as small as 10 mm × 10 mm. The same area was imaged with a smartphone camera (iPhone 11 Pro, Apple) to capture RGB images. The smart neckband was applied on the inside of the human forearm (medial antebrachial cutaneous of the forearm) for 2 hours. As positive controls, 3M electrode (2560, 3M) and CardinalHealth electrode (H124SG, CardinalHealth), were attached on the same area for 2 hours. Images were acquired before and after the experiment for hemoglobin contents comparison. A mechanical linear scan step was performed at 0.25 mm. The data was acquired using a custom-built MATLAB interface. We used a tissue reflectance spectral model to extract key hemodynamic parameters from the ground-truth hyperspectral image. Light propagation in tissue can be modeled om accordance with the theory of radiative transport and robust approximations (e.g., diffusion, Born, and empirical modeling). Specifically, we conducted parameter extractions using an extensively used empirical modeling method. The intensity reflected from a biological sample can be expressed as a function of $\lambda$ in the visible range:

$I_{R}\left( \lambda\right)=\left[ b_{1}\left( \frac{\lambda}{\lambda_{0}} \right)^{b_{2}}+b_{3}\left( \frac{\lambda}{\lambda_{0}} \right)^{-4} \right]\exp\left[ -b_{4}\times\left\{ b_{5}{\times\varepsilon}_{\mathrm{Hb}O_{2}}\left( \lambda\right)+(1-b_{5})\times\varepsilon_{\mathrm{Hb}}\left( \lambda\right) \right\} \right]$ Eq. S2

where $b_{1}$, $b_{2}$, and $b_{3}$ are associated with the scattering (Mie or Rayleigh) contributions at $\lambda_{0}$ = 800 nm, $\varepsilon_{\mathrm{Hb}O_{2}}\left( \lambda\right)$ denotes the absorption coefficient of oxygenated hemoglobin (HbO_2_), $\varepsilon_{\mathrm{Hb}}\left( \lambda\right)$ denotes the absorption coefficient of deoxygenated hemoglobin (Hb), $b_{4}$ is the hemoglobin concentration multiplied by the optical pathlength, and $b_{5}$ is the blood oxygen saturation (sPO_2_). In the study, the hemoglobin contents multiplied by the optical pathlength ($b_{4}$) was used to indicate the level of skin irritation as shown in *SI Appendix* Fig. S4B. all fitting parameters were computed using the simplex search (Nelder-Mead) algorithm.

**Supporting Text 5. Data preprocessing.** The physiological signals harvested from the smart neckband were initially divided into several fixed-length frames to facilitate activity prediction. Once normalized, the signals assembled in the same time instance were concatenated horizontally to create feature vectors associated with specific activities. In the realm of individual activity detection, a single activity was annotated within a given frame out of many, necessitating a multi-class approach whereby an ML or deep learning (DL) algorithm would discern one class from a plethora of others. Conversely, concurrent activities presented a scenario wherein multiple activities coexisted in a solitary data frame. This condition dictated a shift from identifying single activities to predicting each unique activity present in the frame, a strategy realized through the utilization of the Label Powerset algorithm. This tool transformed the multi-class dataset into a multi-label format, a transition that retained label correlation while curtailing the probability of spawning unrealistic predictions. Subsequently, the reconfigured multi-class data were introduced to an ML algorithm known as the RF classifier, setting the stage for model fitting and predictive analysis.

**Supporting Text 6. Model evaluation and comparative analysis.** The training dataset covered a range of activities: individual instances (including stationary, walking, speech, fluid, and food intake) and concurrent permutations (combining walking states with speech, fluid, and food intake). A comprehensive illustration of the activity recognition approach is available in *SI Appendix* Fig. S14A. Furthering the predictive endeavor, Classification and Regression Tree (CART) classifiers were deployed to forecast either a distinct class or a target value, drawing upon a decision tree algorithm (6). The RF (7) is a combination of these CARTs that are denoted as *Tn*:

*T = T_1_(X), T_2_(X), …, T_n_(X)*

where X represents the features for individual CART. The RF leverages a randomly chosen assortment of inputs at each node to formulate a preset array of decision trees. Employing bootstrap aggregation, the RF constructs divergent training subsets from the training samples, each maintaining an identical sample quantity per node, and facilitates a democratic process where each CART endorses the prevalent class within its purview. A concurrence emerges through a majority ruling orchestrated by the RF, a process meticulously detailed in *SI Appendix* Fig. S14B.

Here, training subsets, each containing n number of samples, are generated from the given dataset by bootstrap aggregation. Each training subset utilizes an individual random feature vector (distinguished by variance in color gradient) to train the trees for final voting, thus generating ***n*** number of votes in total. The final prediction was determined by taking the majority vote from all the decisions. To elaborate further, (1) The algorithm starts by creating multiple random subsets of the original dataset through a process called bootstrapping (random sampling with replacement). (2) Each subset is used to train an individual decision tree. This random sampling ensures diversity among the trees. (3) For each subset, a decision tree is constructed using a subset of features at each node. (4) Once all the decision trees are trained, they collectively make predictions on new, unseen data. Each tree votes for a class and the class with the most votes becomes the final prediction. The whole process is demonstrated in *SI Appendix* Fig. S14B, where a pre-determined ***m*** number of inputs were randomly used for the nodes, and each node cast a vote for the most probable class, thus generating a total of ***n*** votes. The RF selects the class with the most votes among the ***n*** number of votes. The redefined problem set derived from the preceding stage found its way to the RF classifier, a move that yielded activity predictions ripe for performance evaluation. The simulation parameters were set to a tree count of 50 and a two-second frame duration. Furthermore, we instituted a comparative analysis of the RF classifier against alternative ML strategies and a traditional CNN-based DL blueprint. For the CNN based approach the dataset was split into 80%–20% for training and testing purpose. Emerging triumphant, the RF classifier outshone the CNN paradigm as well as its rival techniques including k-nearest neighbors (KNN) and support vector machine (SVM), underscoring its adeptness in tackling complex classification undertakings (*SI Appendix* Table S3). In pursuit of impeccable accuracy, we initiated every simulation, excluding the DL method, a total of ten times, deploying a 10-fold CV to endorse the results steadfastly. The experiments were conducted on NVIDIA Geforce GTX 1080 Ti.


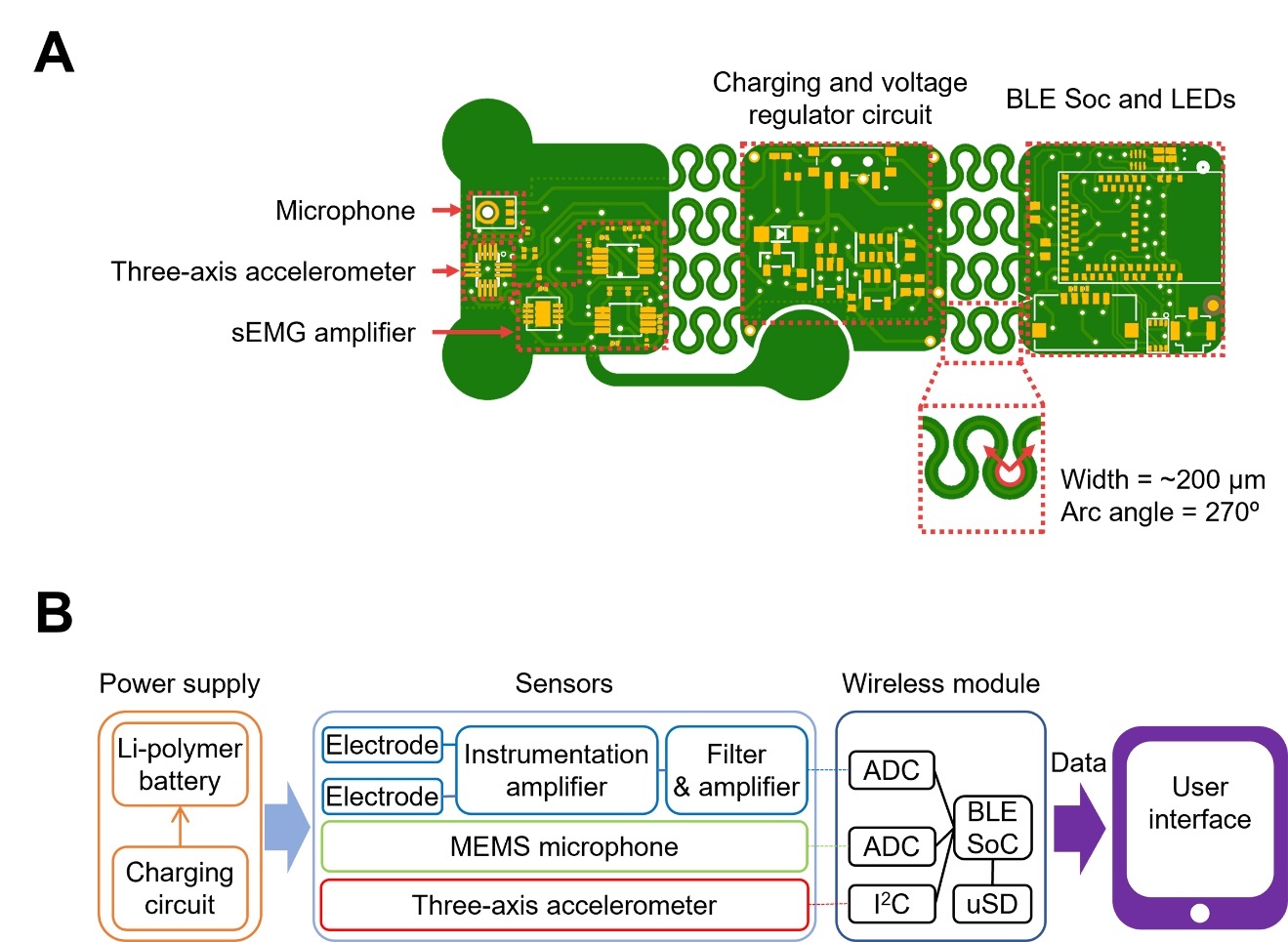


Fig. S1. Layout of the flexible printed circuit board (fPCB) and block diagram for the smart neckband. (A) Schematic illustration of the design featuring three interconnected layers in a serpentine pattern. The first layer houses a microphone, a three-axis accelerometer, and an sEMG amplifier. The second layer contains circuits for charging and voltage regulation. The third layer incorporates the Bluetooth Low-Energy (BLE) system-on-chip (SoC) and LED indicators. (B) Block diagram for the smart neckband detailing the power supply, sensors, and wireless module. The power supply with a Li-polymer battery and charging circuit feeds into sensors including a custom-designed sEMG amplifier, a MEMS microphone, and a three-axis accelerometer. Analog signal is acquired by ADCs, with data transmission via BLE SoC to the user interface.


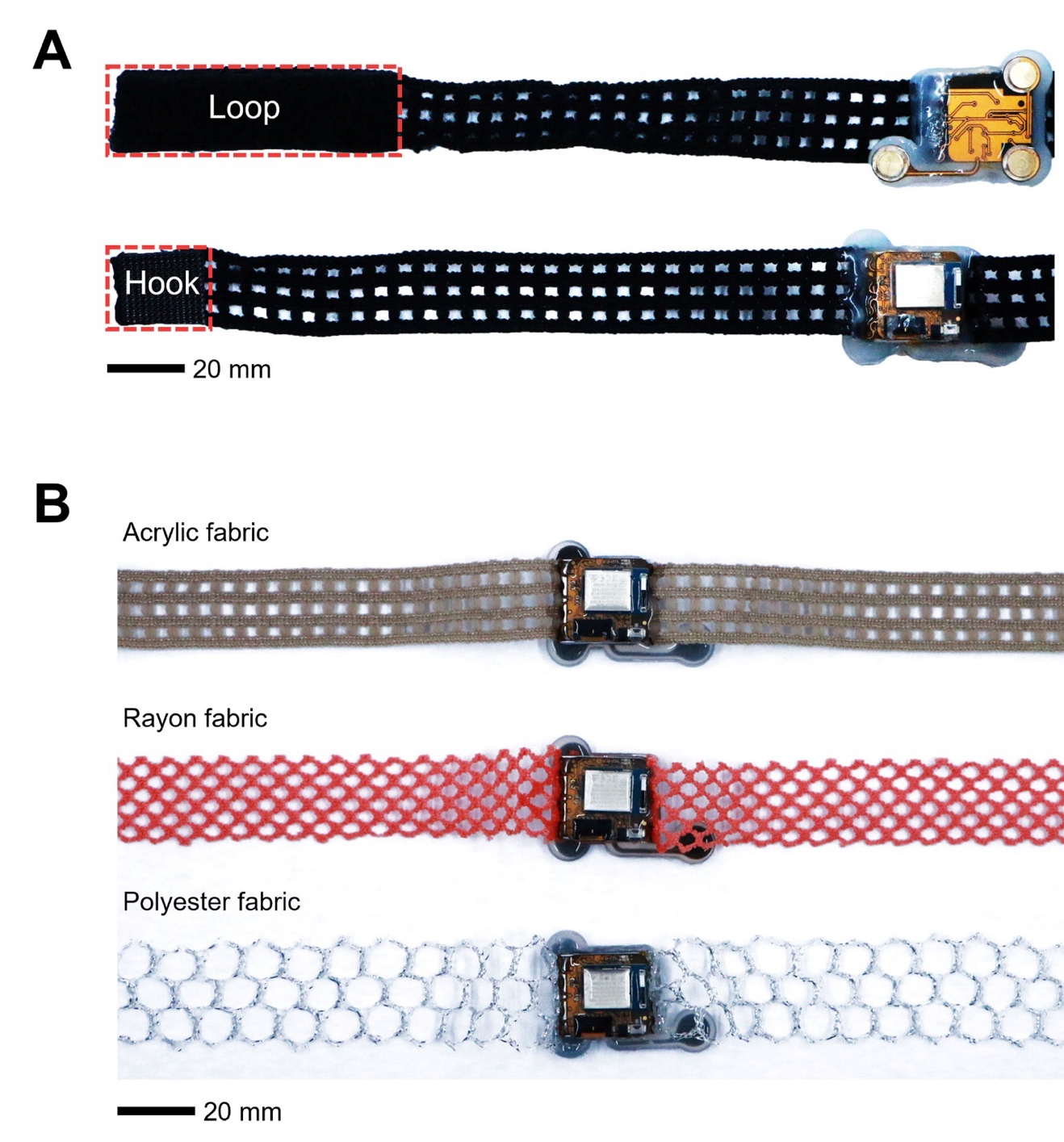


Fig. S2. Adjustable hook-and-loop strap and textile band selection. (A) Photos of the hook-and-loop strap designed to accommodate varying neck circumferences. (B) Photos of commercial neckbands in a variety of mesh sizes, shapes, colors, and materials—including acrylic, rayon, and polyester—to cater to diverse neck sizes and textile preferences.


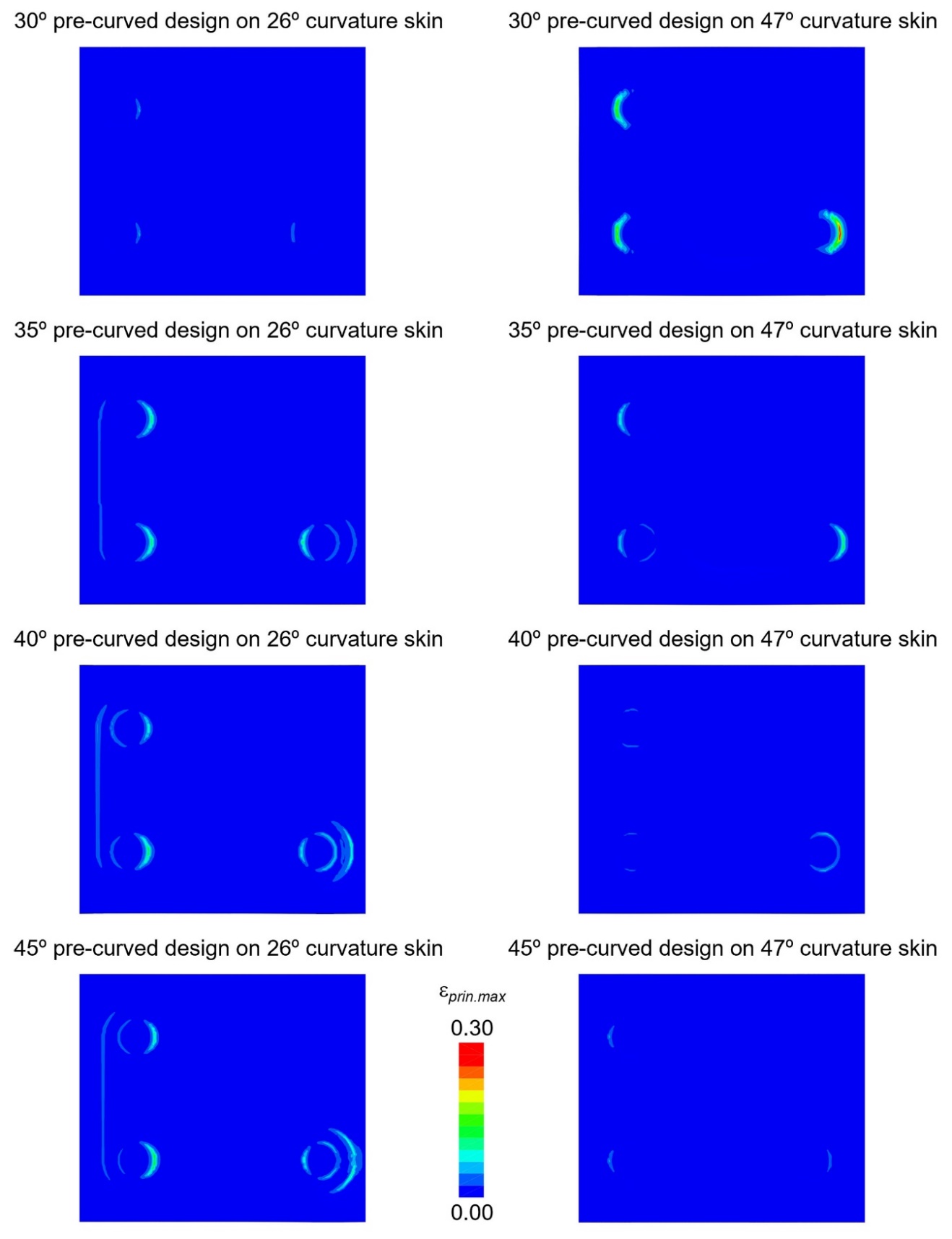


Fig. S3. Strain distribution with a pre-curved device placed on curved skin with varying angles. FEA results of the strain distribution caused by attaching a pre-curved device (30º, 35º, 40º, 45º) to skin with curvatures of 26º and 47º.


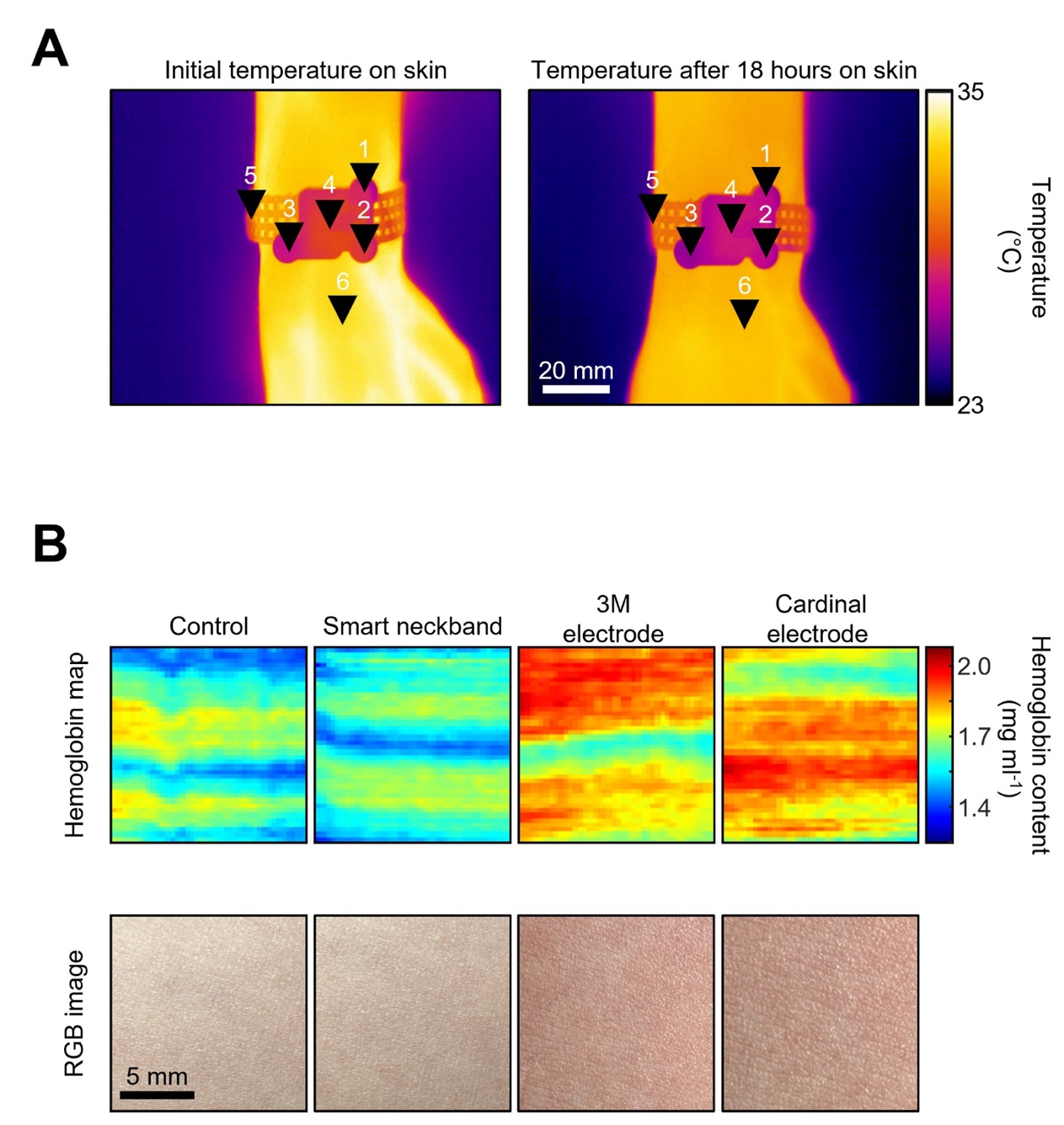


Fig. S4. Temperature distribution on skin and skin irritation test. (A) Infrared (IR) images of temperature variations on both the sensor module surface and adjacent skin areas over an 18-hour period. (B) Maps of hemoglobin distribution and corresponding RGB images for the control, smart neckband, and commercial electrodes on human skin.


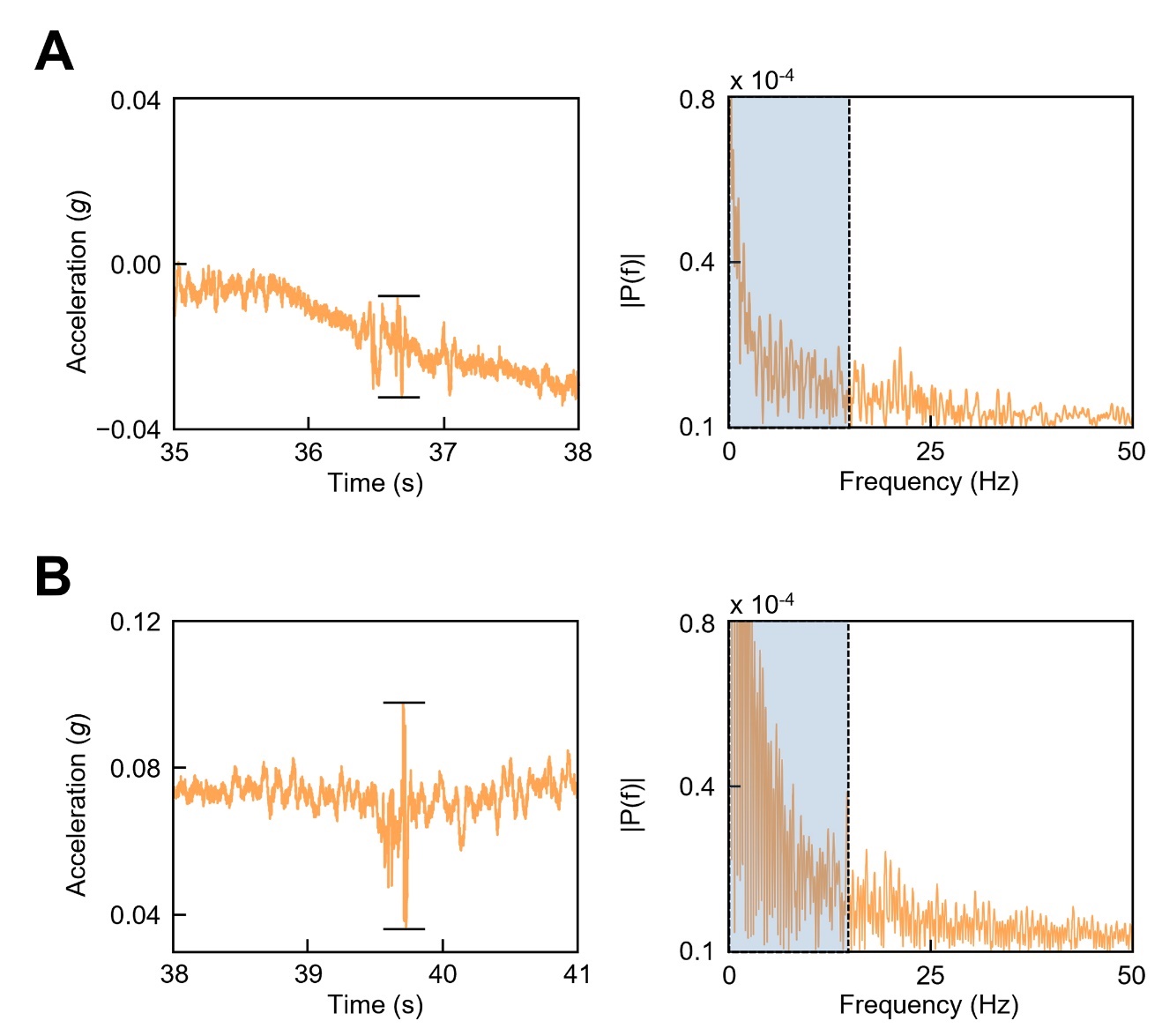


Fig. S5. Comparison of fluid intake according to viscosity while stationary state. (A) Y-axis acceleration signal (left), and a fast Fourier transform signal (right) when water intake. (B) Y-axis acceleration signal (left), and a fast Fourier transform signal (right) when high-viscosity liquid intake.


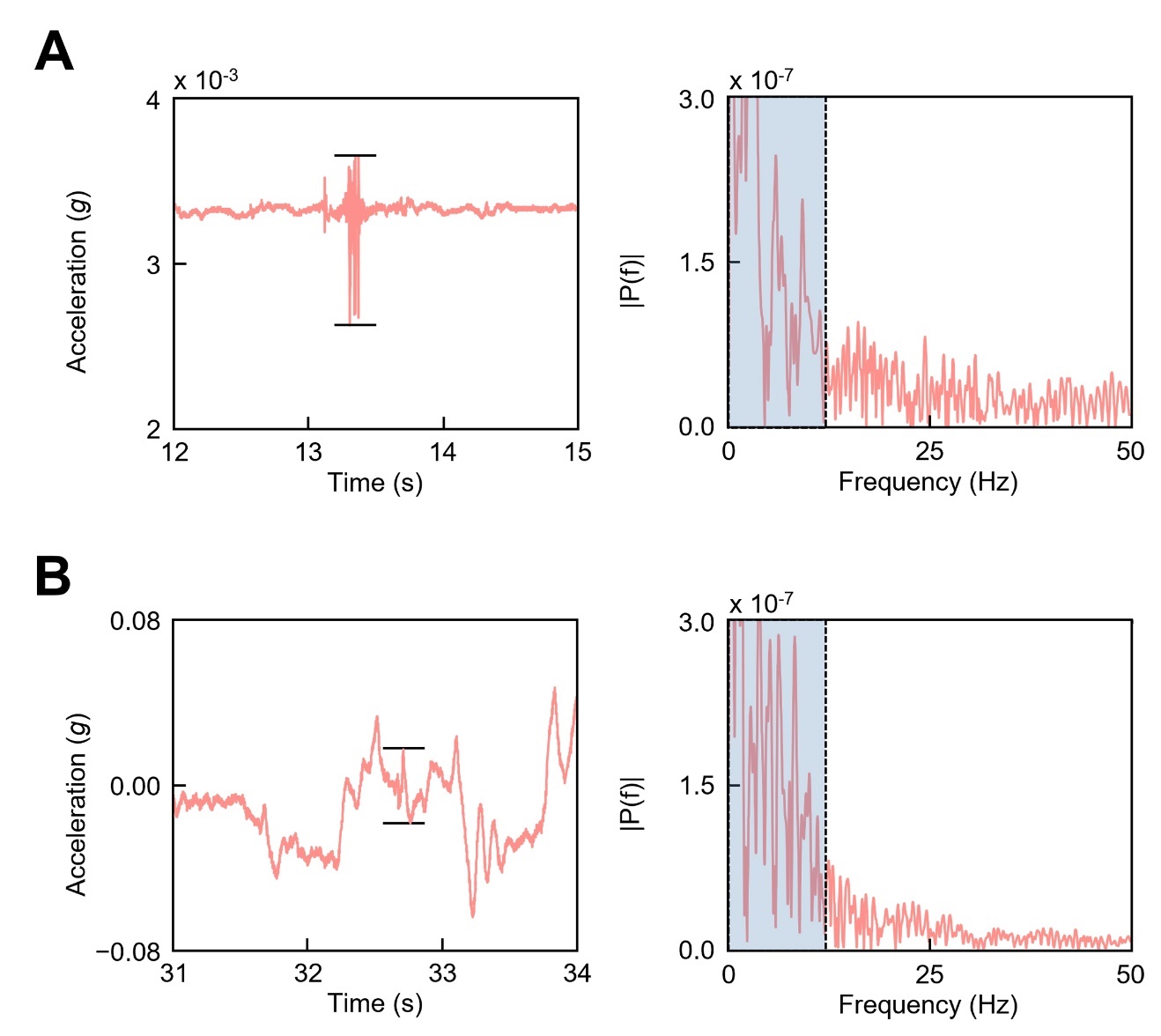


Fig. S6. Comparison of fluid intake according to viscosity while walking state. (A) X-axis acceleration signal (left), and a fast Fourier transform signal (right) when water intake. (B) X-axis acceleration signal (left), and a fast Fourier transform signal (right) when high-viscosity liquid intake.


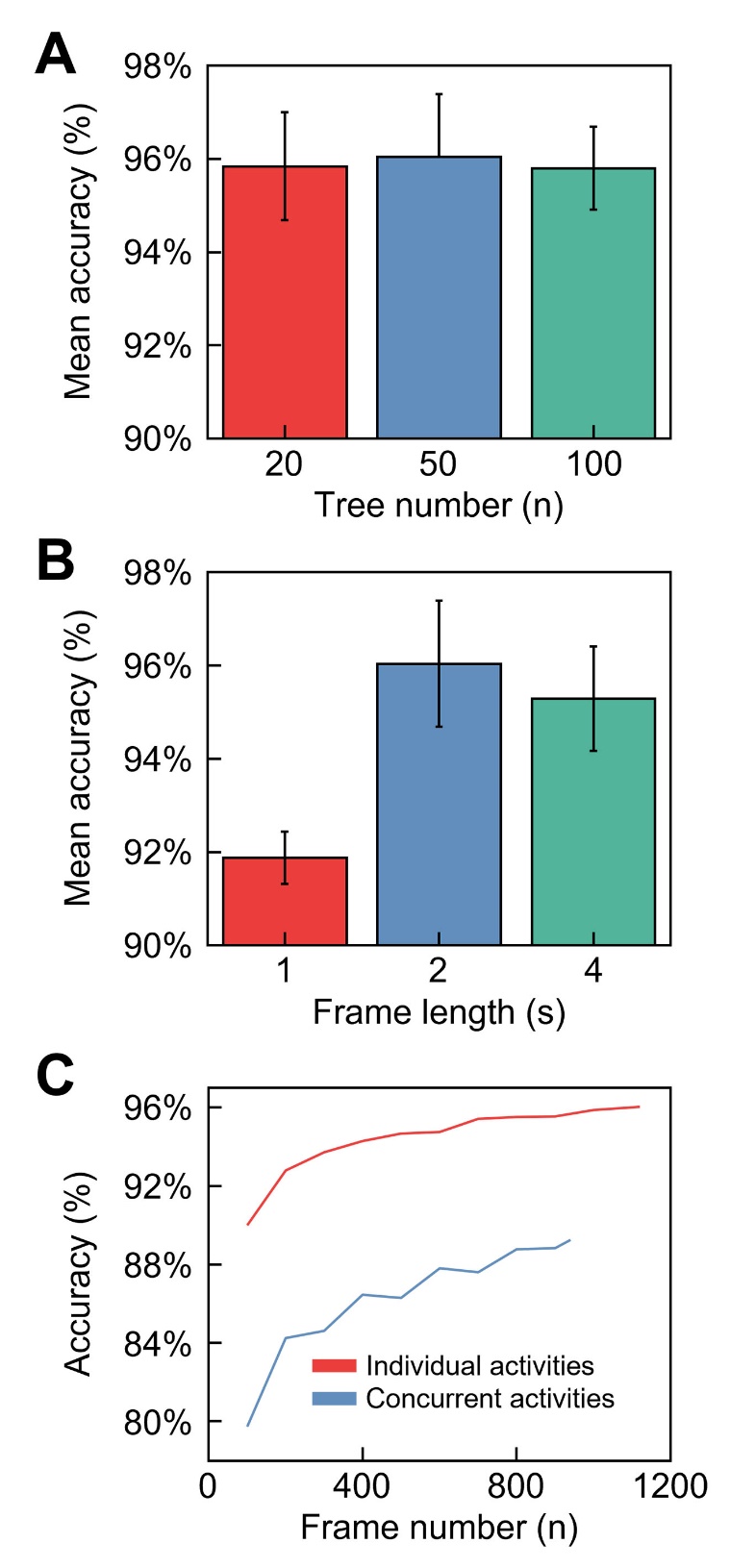


Fig. S7. Random forest (RF) classifier parameter analysis. (A) Assessment of classification accuracy with tree numbers, ranging from 20 to 100 trees. (B) Classification accuracy assessment based on varying frame lengths, ranging from 1 to 4 seconds. (C) Evaluation of its performance based on varying dataset sizes.


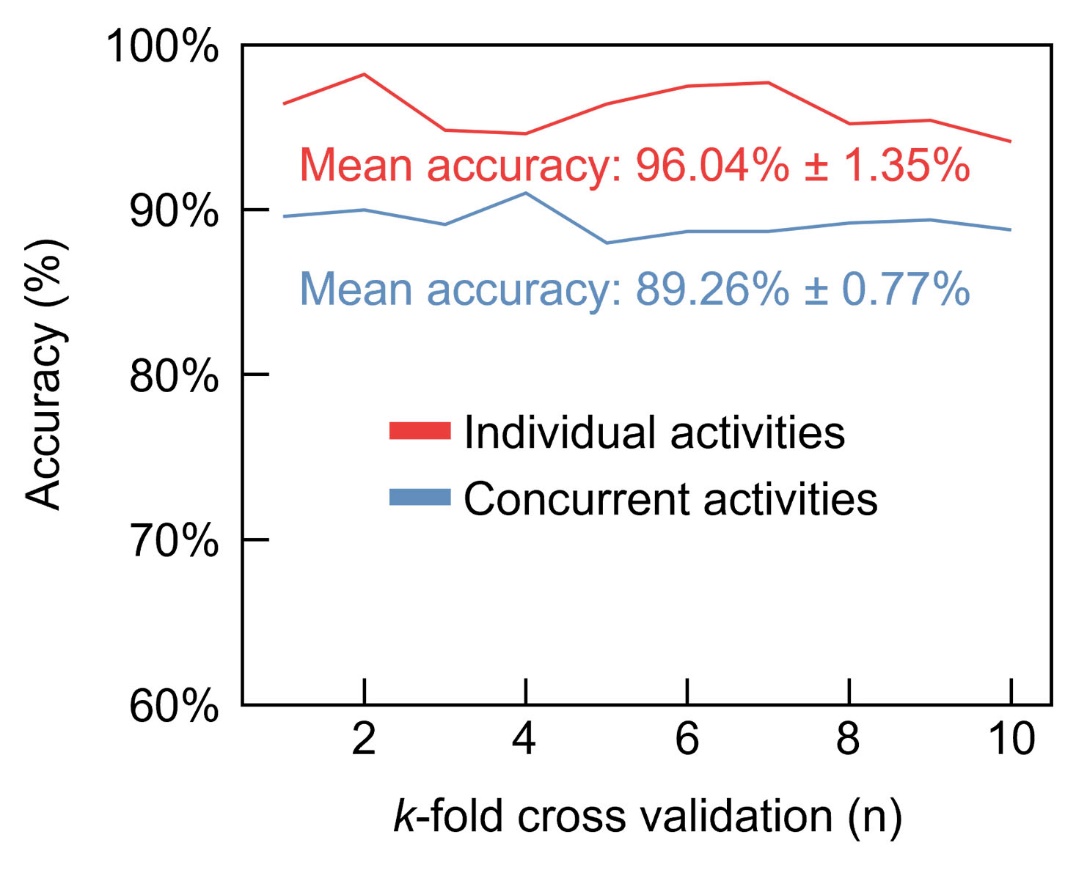


Fig. S8. Analysis of RF classifier accuracy based on variations in '*k*'. Results of changes in accuracy and mean accuracy as determined by *k*-fold cross-validation.


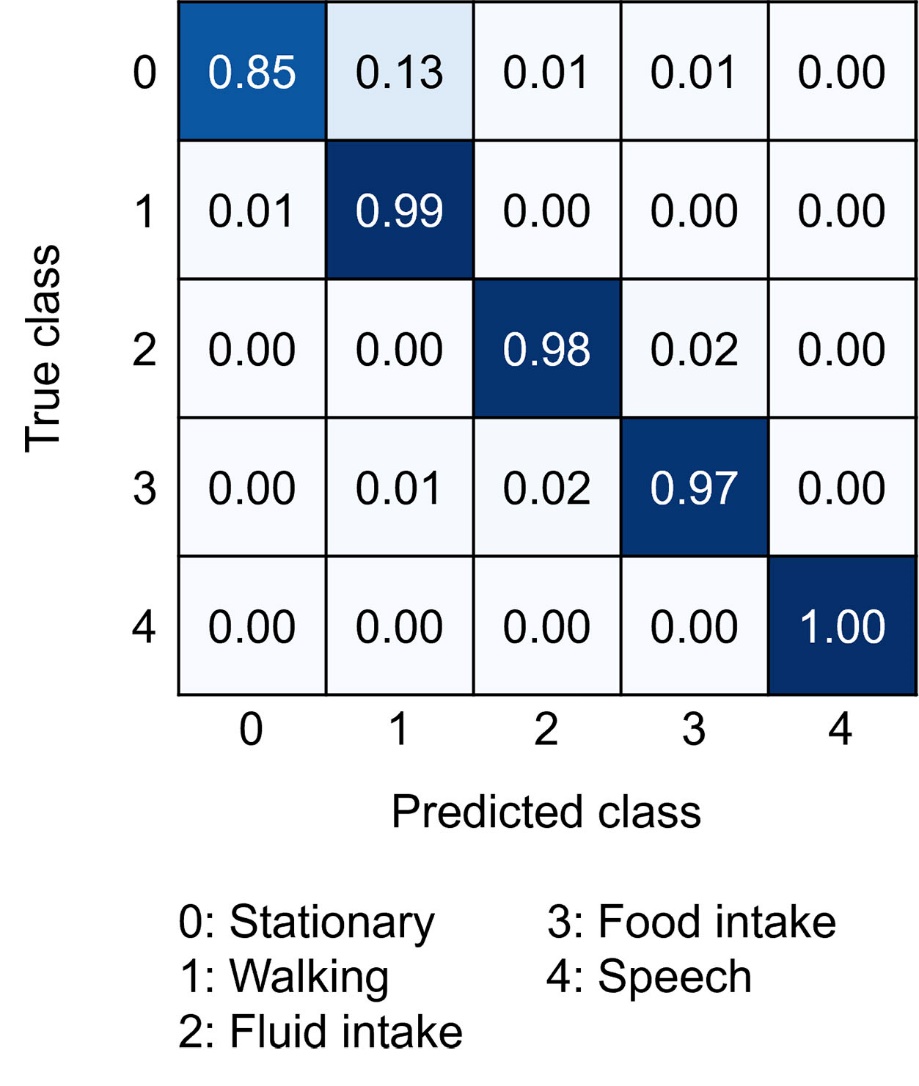


Fig. S9. Confusion matrix of individual activities. Results of classifying five activity types: stationary, walking, fluid intake, food intake, and speech.


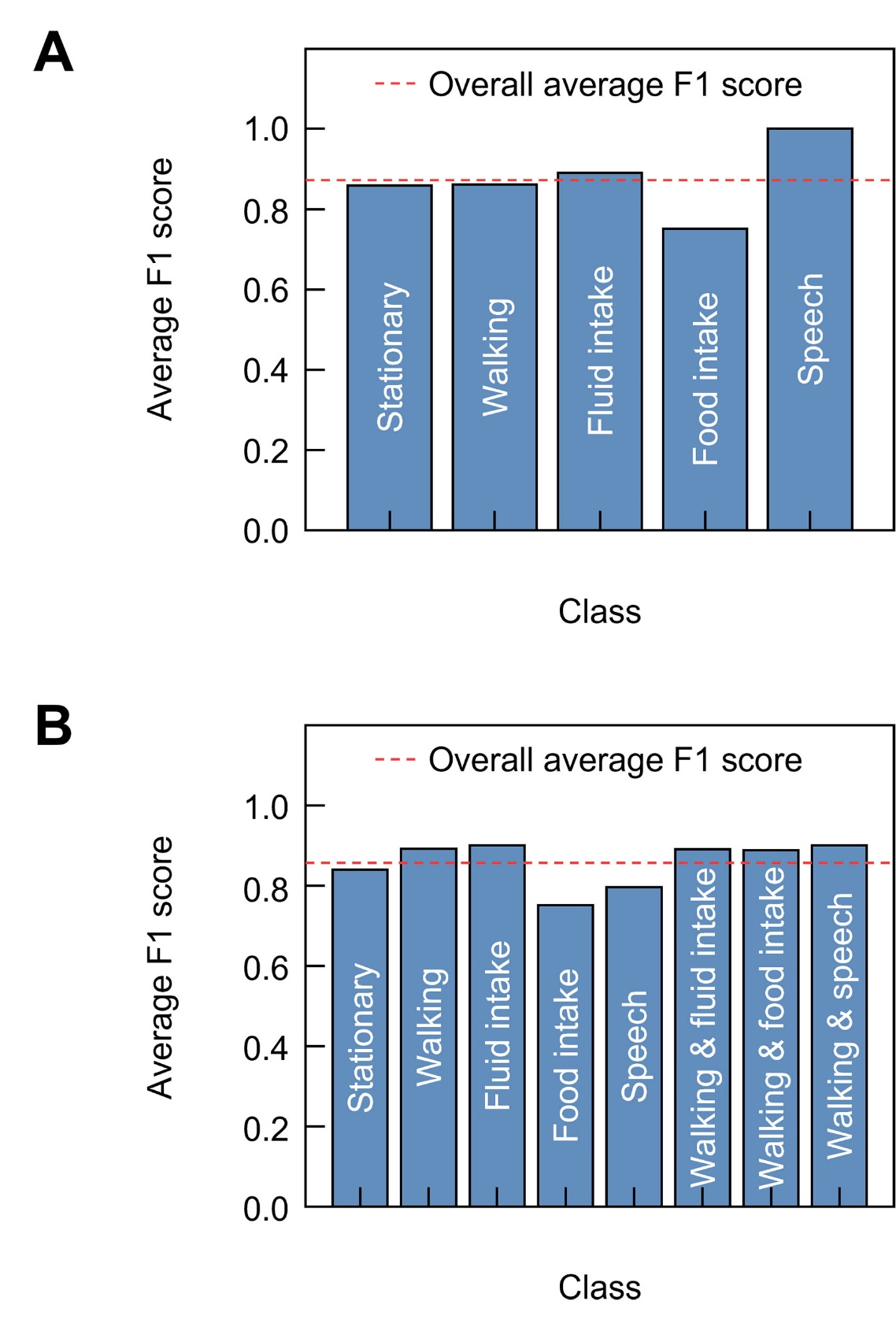


Fig. S10. Comparison of average F1 scores across classes for individual and concurrent activities. (A) F1 scores for individual activities, with the classes including stationary, walking, fluid intake, food intake, and speech, with an overall average F1 score of 0.873. (B) F1 scores for concurrent activities, representing classes such as stationary, walking, fluid intake, food intake, speech, walking & fluid intake, walking & food intake, and walking & speech, with an overall average F1 score of 0.857.


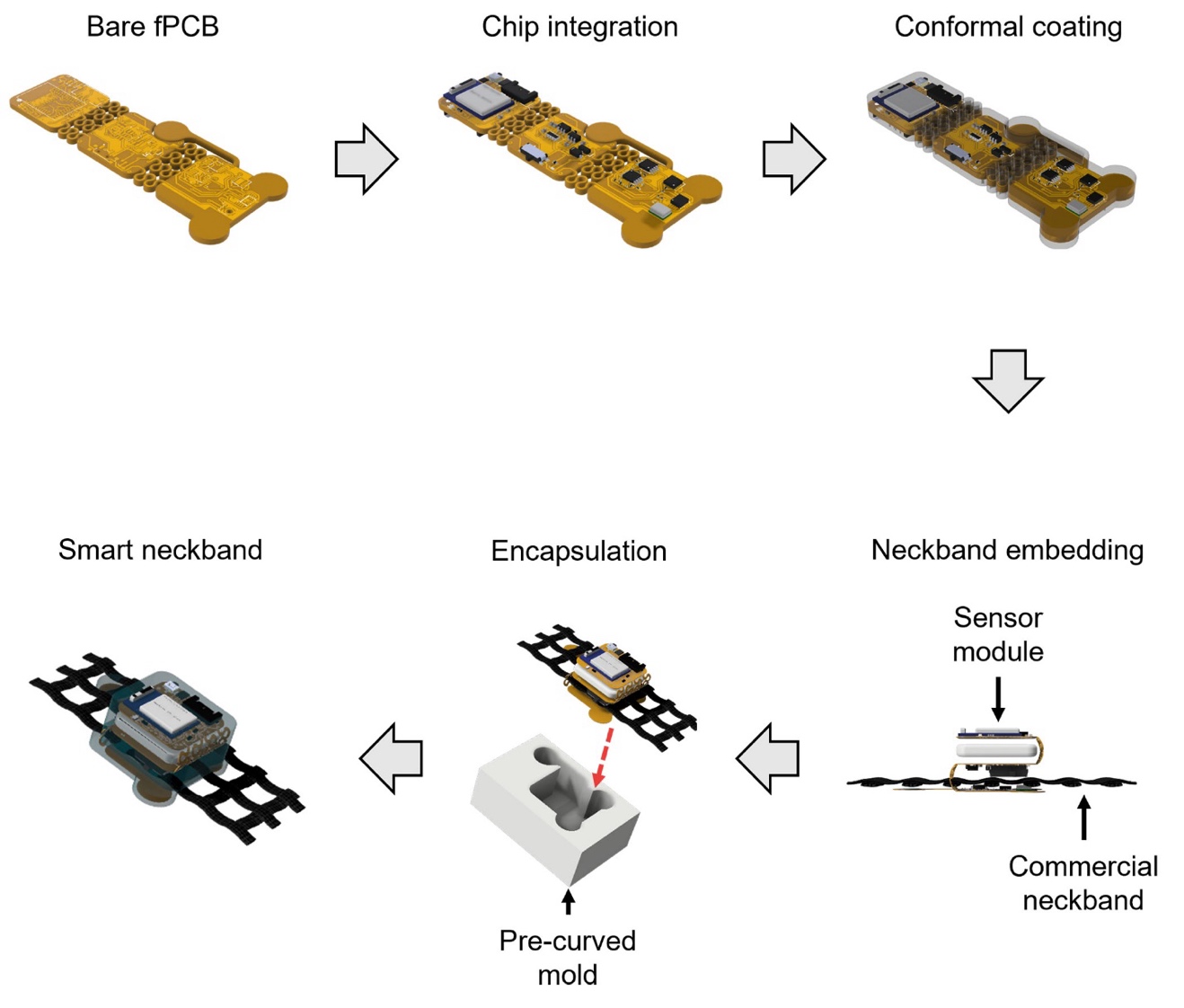


Fig. S11. Smart neckband fabrication steps. Schematic illustration of the step-by-step fabrication process: First, the sensor module was fabricated by soldering electronic components onto the bare flexible printed circuit board. Next, a conformal coating was applied to waterproof and enhance the robustness of both the electronic components and the substrate. Finally, the finalized sensor module was integrated into a commercial neckband and underwent a soft, waterproof encapsulation process.


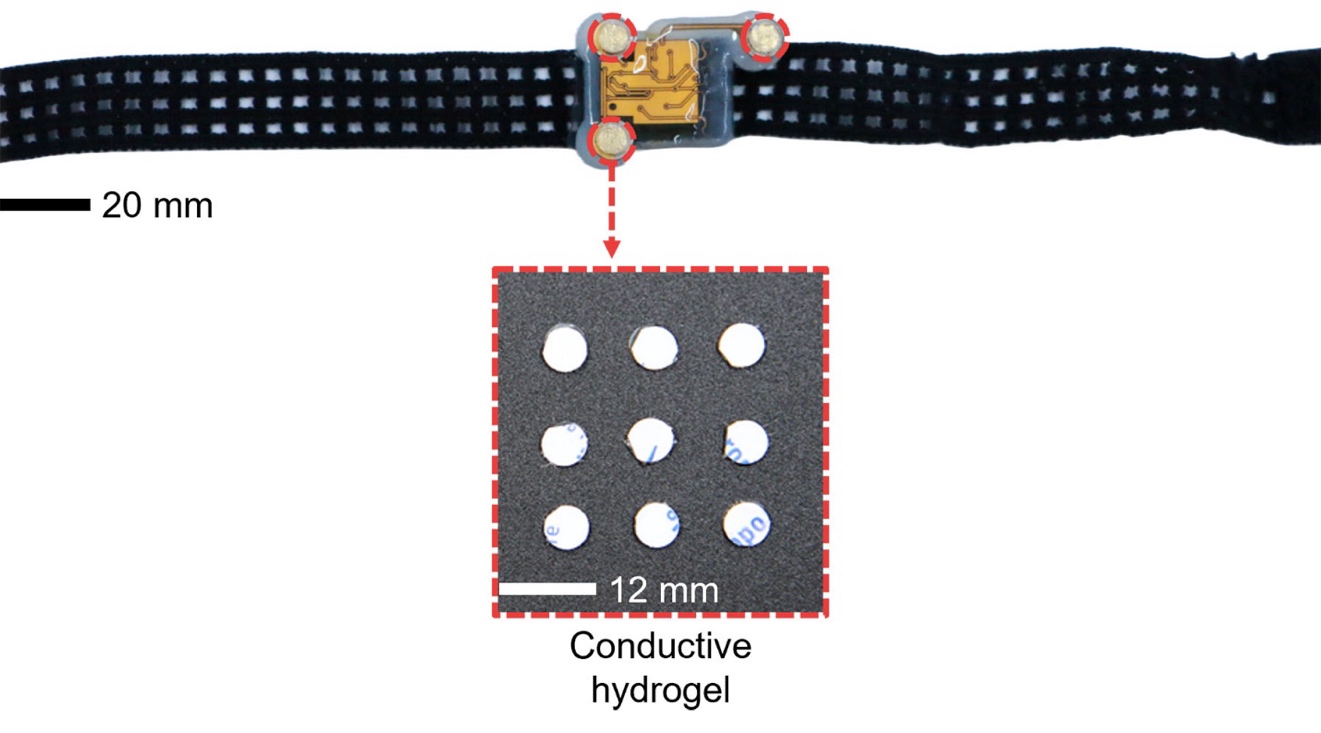


Fig. S12. Conductive hydrogels. Photos of the conductive hydrogels used to couple the sEMG electrodes to the skin, facilitating the measurement of EMG signals.


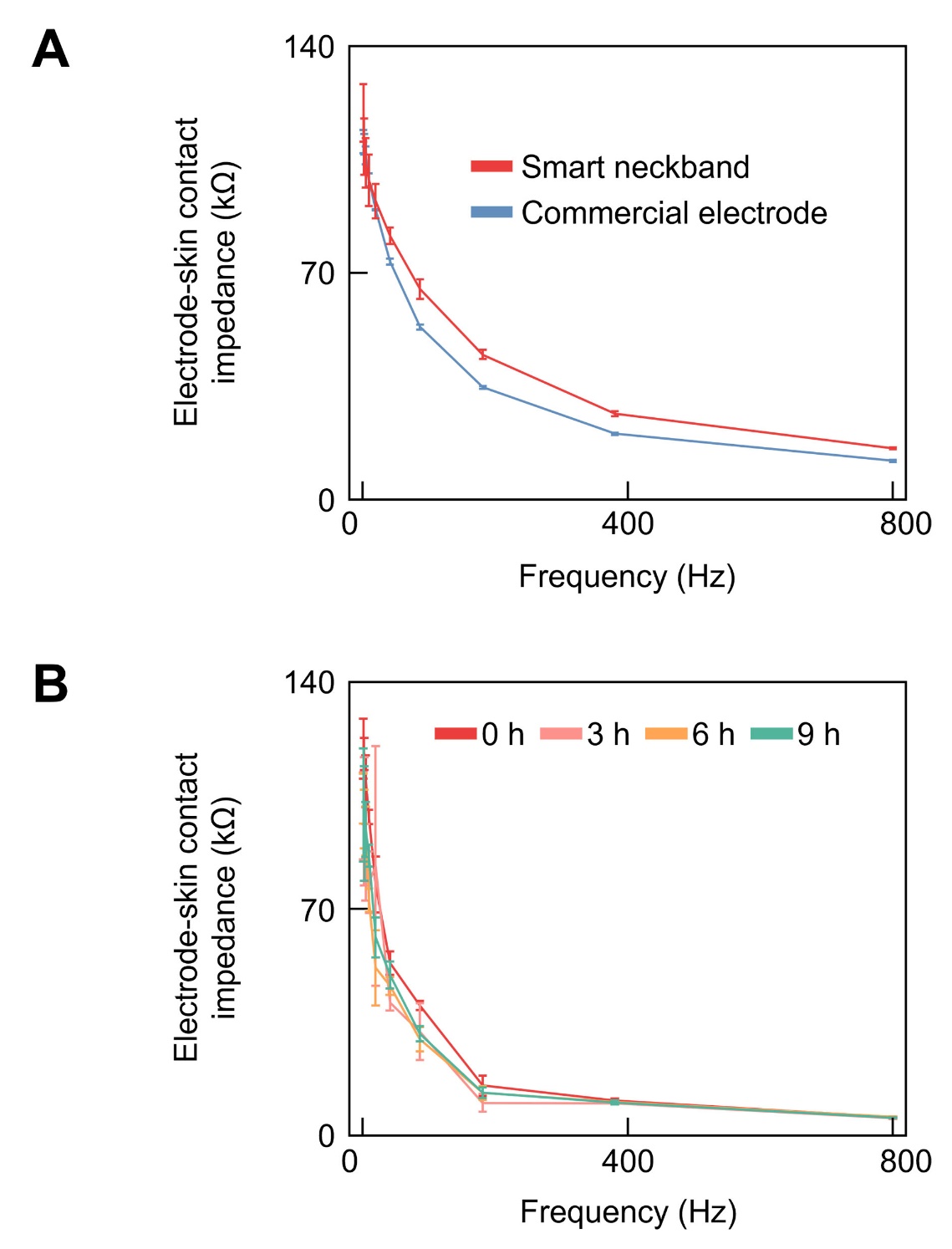


Fig. S13. Impedance measurement comparison. (A) Comparison of the electrode-skin contact impedances between the sEMG electrodes of the smart neckband (red) and commercial recording electrodes (blue). (B) Analyzing the impedance at the electrode-skin interface for smart neckband sEMG electrodes following air exposure periods of 0, 3, 6, and 9 hours.


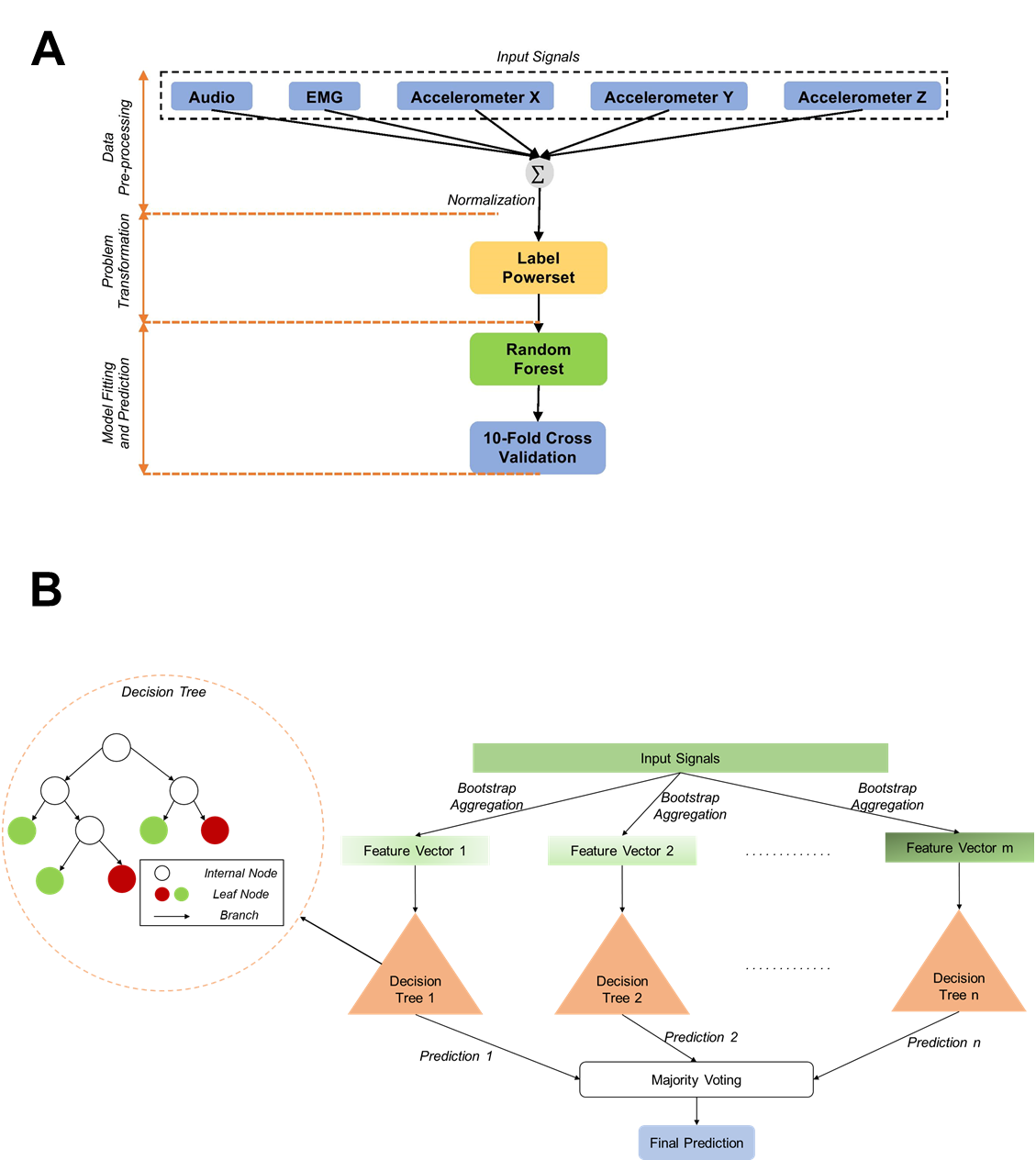


Fig. S14. Methodology of random forest classification. (A) Comprehensive approach for activity classification. (B) Detailed process for the random forest classifier.

Table S1. Comparative analysis of dietary intake detection with previous studies. Summary of relevant studies that utilized wearable devices. 'N/R' denotes 'not reported'.


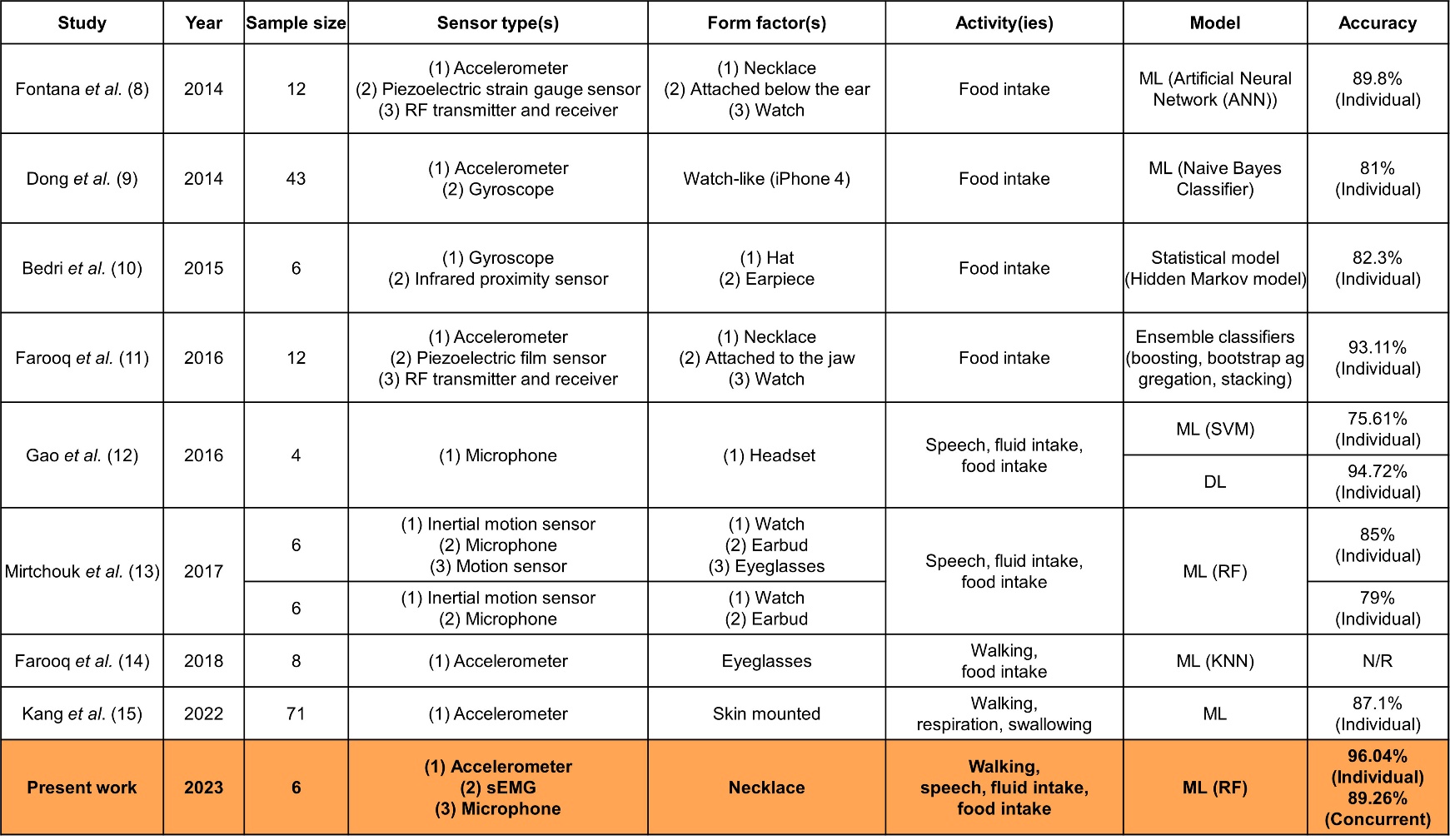


Table S2. Evaluation of mean accuracy across each activity. Mean classification accuracy for activities such as walking, speech, and fluid and food intake, considering them both as individual and concurrent activities.


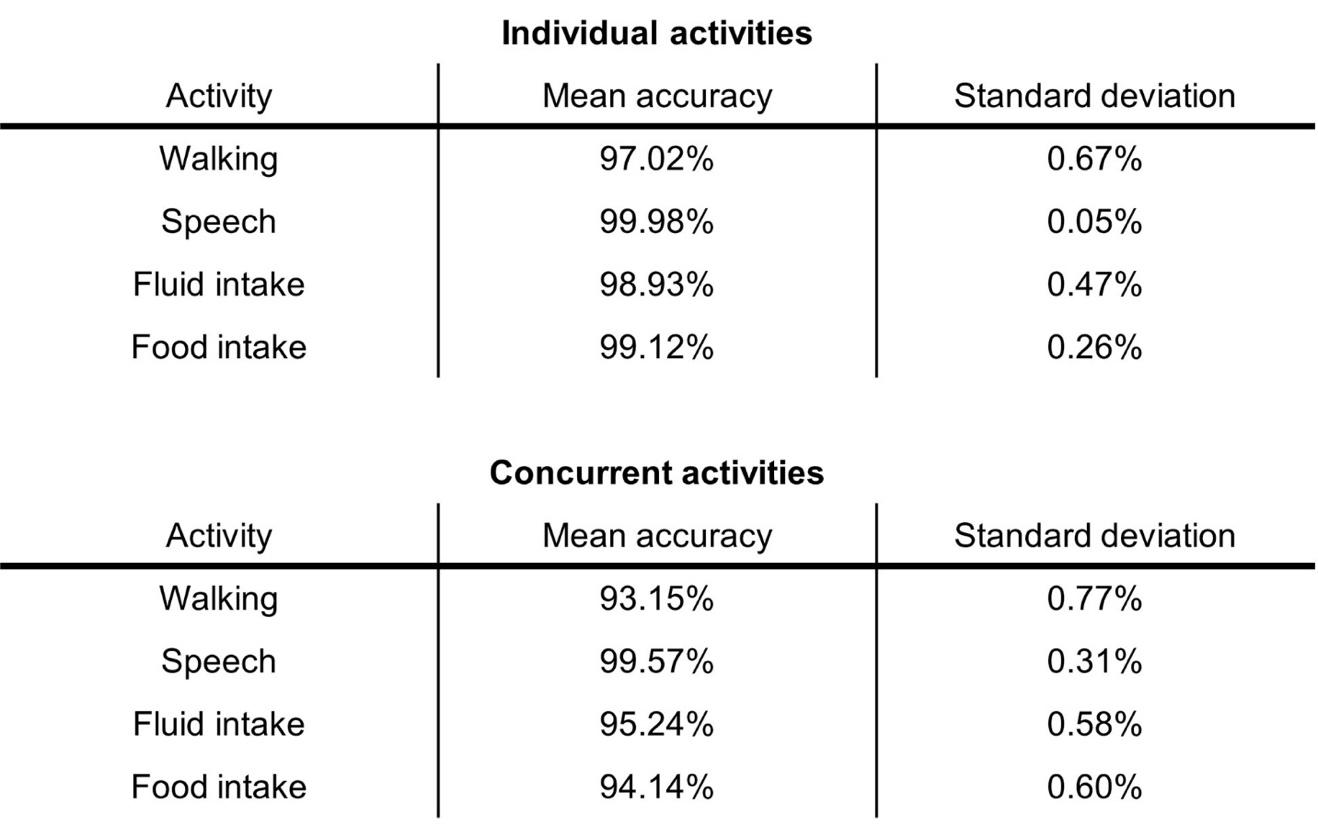


Table S3. Performance evaluation across various classifiers. Average classification accuracy by classifier types, including K-nearest neighbor (KNN), support vector machine (SVM), convolutional neural network (CNN), and random forest (RF). Abbreviations used: ML - Machine learning; DL - Deep learning.


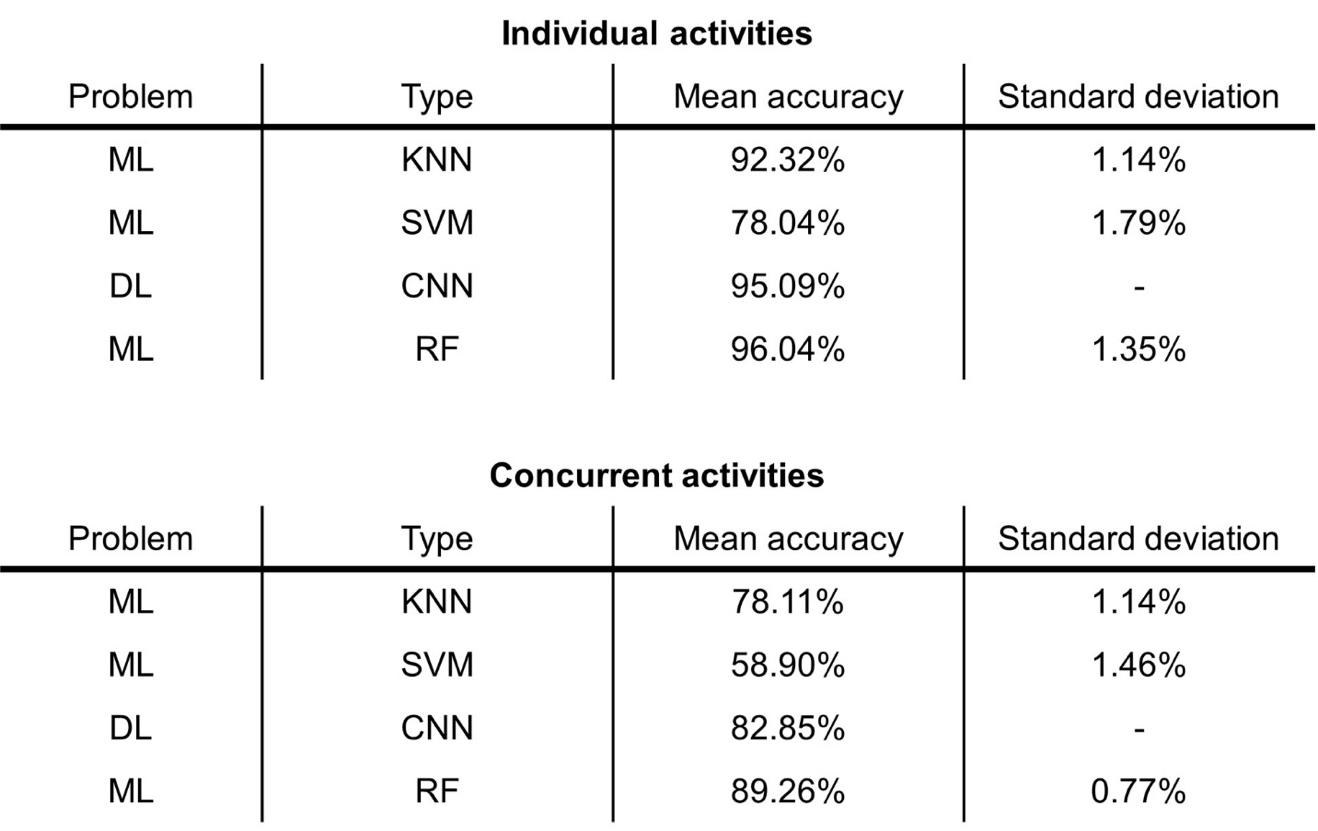


Movie S1 (separate file). Overview of activities with real-time data transmission. Display of real-time data plotted in sequential order, covering stationary, speech, fluid intake, food intake, walking, speech during walking, fluid intake during walking, and food intake during walking.

Movie S2 (separate file). Mechanical simulation of strain distribution in interconnectors. Illustration highlighting the strain variations experienced by the interconnectors of the sensor module during folding.

Movie S3 (separate file). Mechanical simulation of strain distribution in the fPCB substrate. Depiction of strain variations within the substrate of the sensor module throughout the pre-curving process.

Movie S4 (separate file). Mechanical simulation demonstrating strain variations under stretching and twisting. Display of the strain experienced by the sensor module during sequential actions, from 0%–60% stretching, 0°–180° twisting, to 0%–60% stretching post 180° twisting.

Movie S5 (separate file). Vibration and waterproof tests. Depiction of vibration test at 2 Hz for the sensor modules measuring 20 × 20 mm² and 20 × 40 mm², followed by a waterproof test for the smart neckband.

**SI References**

1. G. Zhang *et al.*, Intermittent Theta-Burst Stimulation Over the Suprahyoid Muscles Motor Cortex Facilitates Increased Degree Centrality in Healthy Subjects. *Front. Hum. Neurosci*. **14**, 516699 (2020).
2. P. L. Allan, G. M. Baxter, M. J. Weston, “Ultrasound of the neck” in *Clinical Ultrasound* (Elsevier Health Sciences, ed. 3, 2011), pp. 890–919.
3. Y.-z. Yan *et al.*, Normal radiological anatomy of thyroid cartilage in 600 Chinese individuals: Implications for anterior cervical spine surgery. *J Orthop. Surg. Res*. **13**, 1–8 (2018).
4. G. D. de Melo *et al.*, COVID-19-related anosmia is associated with viral persistence and inflammation in human olfactory epithelium and brain infection in hamsters. *Sci Transl. Med*. **13**, 8396 (2021).
5. Y. Ji *et al.*, mHealth hyperspectral learning for instantaneous spatiospectral imaging of hemodynamics. *PNAS Nexus*. **2**, 1–15 (2023).
6. B. A. Martínez *et al.*, Machine learning reveals distinct gene signature profiles in lesional and nonlesional regions of inflammatory skin diseases. *Sci. Adv*. **8**, eabn4776 (2022).
7. L. Breiman, Random forests. *Machine learning*. Springer **45**, 5–32 (2001).
8. J. M. Fontana, M. Farooq, E. Sazonov, Automatic ingestion monitor: A novel wearable device for monitoring of ingestive behavior. *IEEE Trans. Biomed. Eng*. **61**, 1772–1779 (2014).
9. Y. Dong *et al.*, Detecting periods of eating during free-living by tracking wrist motion. *IEEE J. Biomed. Health. Inform*. **18**, 1253–1260 (2014).
10. A. Bedri *et al.*, In *2015 ACM International Conference on Multimodal Interaction (ICMI)* (ACM 2015).
11. M. Farooq, E. Sazonov, In *38th Annual International Conference of the IEEE Engineering in Medicine and Biology Society (EMBS)* (IEEE 2016).
12. Y. Gao *et al.*, In *2016 IEEE 1st International Conference on Connected Health: Applications, Systems and Engineering Technologies (CHASE)* (IEEE 2016).
13. M. Mirtchouk *et al.*, Recognizing Eating from Body-Worn Sensors. *Proc. ACM Interact. Mob. Wearable Ubiquitous Technol*. **1**, 1–20 (2017).
14. M. Farooq, E. Sazonov, Accelerometer-Based Detection of Food Intake in Free-Living Individuals. *IEEE Sens. J*. **18**, 3752–3758 (2018).
15. Y. J. Kang *et al.*, Soft skin-interfaced mechano-acoustic sensors for real-time monitoring and patient feedback on respiratory and swallowing biomechanics. *npj Digit. Med*. **5**, 1–13 (2022).
